# The Tec kinase ITK is essential for ILC2 survival and epithelial integrity in the intestine

**DOI:** 10.1038/s41467-019-08699-9

**Published:** 2019-02-15

**Authors:** Hyoung-Soo Cho, Andrea Reboldi, Jason A. Hall, Leslie J. Berg

**Affiliations:** 1 Department of Pathology, University of Massachusetts Medical School, Worcester, MA 01605 USA; 2 The Kimmel Center for Biology and Medicine of the Skirball Institute, New York University School of Medicine, New York, NY 10016 USA

## Abstract

Innate lymphoid cells (ILC) are lymphocytes that lack an antigen-specific receptor and are preferentially localized in non-lymphoid tissues, such as mucosal barriers. In these locations ILC respond to tissue perturbations by producing factors that promote tissue repair and improve barrier integrity. We show that mice lacking the Tec kinase ITK have impaired intestinal tissue integrity, and a reduced ability to restore homeostasis after tissue damage. This defect is associated with a substantial loss of Type 2 ILC (ILC2) in the intestinal lamina propria. Adoptive transfer of bone marrow ILC2 precursors confirms a cell-intrinsic role for ITK. Intestinal ILC2 numbers in *Itk*^*-/-*^ mice are restored by the administration of IL-2 complexes, also leading to improved intestinal tissue damage repair. Reduced Bcl-2 expression in intestinal *Itk*^*-/-*^ ILC2 is also restored to WT levels after IL-2 complex treatment, indicating a tissue-specific role for ITK in ILC2 survival in the intestine.

## Abstract (web summary)

Signals mediating tissue-specific control of immune cell fate and function remain largely uncharacterized. Here the authors uncover a requirement of ITK, a kinase mediating TCR signaling, for IL-2-dependent ILC2 cell survival specifically in the intestine.

## Introduction

Innate lymphoid cells are one of a subset of lymphocytes that lack an antigen-specific receptor; yet, they produce effector molecules shared with CD4^+^ T cells^1–4^. Whereas adaptive lymphocytes are abundant in lymphoid tissues, ILC are preferentially localized in non-lymphoid tissues, most notably at mucosal barriers^5^. Their positioning at mucosal surfaces confers a strategic advantage to ILC, allowing them to respond promptly to bacterial or viral infections^6–9^. ILC are thought to be important in regulating mucosal barriers by triggering epithelial cell growth or modulating tissue integrity and homeostasis^5,10^. ILC subsets can be categorized into cytotoxic ILC and non-cytotoxic helper-like ILC. Each helper-like ILC subset expresses a key transcription factor that regulates a distinct cytokine profile corresponding to their adaptive CD4^+^ T cell counterparts: T-bet for ILC1, GATA-3 for ILC2, and RORγt for ILC3^1,2,4^.

ILC2 were first identified in mesenteric lymphoid clusters and were later shown to be scattered in the lung and intestinal lamina propria (LP)^11–13^. ILC2 express a set of surface markers (e.g., CD90, CD127, CD25, IL-25R, and IL-33R) along with the signature transcription factor, GATA-3^1,3,14^. ILC2 are known to be activated by alarmins, such as IL-25, IL-33, and thymic stromal lymphopoietin (TSLP)^11–13,15,16^. Upon stimulation by these cytokines, ILC2 produce IL-5, IL-9, IL-13, and amphiregulin (Areg), which are important effector molecules in responses to helminths in the intestine and promote repair of tissue damage caused by virus infections in the lung^6,17,18^. In addition, IL-2 regulates ILC2 production of IL-5 and IL-9, and IL-2/anti-IL-2 complexes (IL-2c) are known to induce in vivo proliferation of ILC2^19,20^.

ILC emerge from their lymphoid progenitors in the fetal liver and adult bone marrow (BM) and disseminate to various tissues^21,22^. ILC precursors express integrin α4β7, the receptor for mucosal vascular addressin cell adhesion molecule 1 (MAdCAM-1), an integrin ligand expressed by gut-associated endothelial cells^23^. Additionally, ILC precursors express CCR9, a key homing molecule that guides cells to intestinal tissues. Previous studies showed that retinoic acid (RA) upregulates the expression of integrin α4β7 and CCR9 in ILC1 and ILC3 for gut-homing^24^. However, BM ILC2 precursors (ILC2P) are programmed to express these gut-homing receptors, which promote direct gut-homing of ILC2P in an RA-independent manner^24^. In addition to gut-homing, ILC2 dissemination also requires efficient egress of ILC2P from the BM, a process regulated by IL-33^25^. Thus, ILC2 trafficking to peripheral sites is a cooperative process combining successful egress with proper tissue homing.

Despite a lack of antigen-specific receptors, ILC express a series of T-cell receptor (TCR) components, such as LAT, LCK, ICOS, and the Tec family kinase ITK^22,23,26–28^. Transcriptome analysis revealed that ILC have more similarities with T cells than with other adaptive lymphocytes^29^, but the function of TCR components in ILC has not been characterized. Interestingly, Shih et al. recently reported that ITK and IRF4, a TCR downstream transcription factor, were found among the most highly upregulated genes in ILC2^30^. Consistently, RNA-Seq data from the Immunological Genome Consortium (www.immgen.org) shows that *Itk* expression is highly elevated in intestinal ILC2 compared with other ILC subsets in that tissue; in addition, a recent study reports that ILC2 isolated from a variety of tissue sites all express substantial amounts of *Itk* mRNA^31^. Interestingly, ITK is also known to be important for CD4^+^ T-cell migration to the intestine^32^. However, the role of ITK in type 2 innate lymphoid cells has not previously been assessed.

Here, we examine the function of ITK in ILC2 in the intestine. We show that *Itk*^*-−/−*^ mice display a tissue-specific loss of ILC2 in the intestine but not other sites. While *Itk*^*−/−*^ mice have neither deficiency of BM ILC2P nor of gut-homing receptor expression on ILC2, adoptively transferred *Itk*^*−/−*^ ILC2 could not be recovered in the intestine of *Rag1*^*−/−*^*Il2rg*^*-−/−*^ hosts, indicating a cell-intrinsic defect in *Itk*^*−/−*^ ILC2. Intestinal tissue damage following DSS treatment is more severe in *Itk*^*−/−*^ mice; however, injection of IL-2c into *Itk*^*−/−*^ mice restores intestinal ILC2 numbers and improves the response of *Itk*^*−/−*^ mice to DSS. Overall, our data indicate that ITK is essential for ILC2 tissue homeostasis in the intestine and for intestinal barrier integrity.

## Results

### ITK deficiency leads to a steady-state defect in gut ILC2

**Fig. 1:**
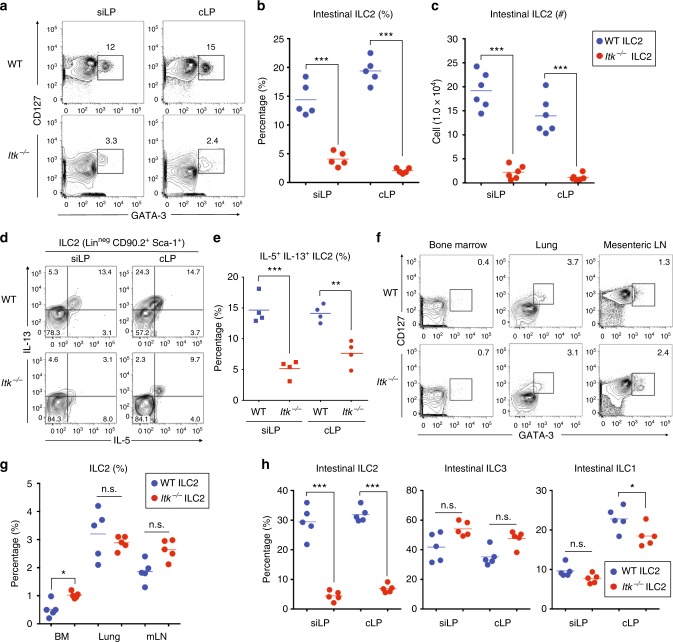
*Itk*^*−/−*^ mice have a gut-specific reduction in ILC2. **a**–**c** The proportions of ILC2 (GATA-3^+^ CD127^+^) in the lamina propria of the small (siLP) or large intestine (cLP) from naïve WT and *Itk*^*−/−*^ mice are shown after gating on lineage-negative cells (CD3ε^−^ CD19^−^ TCRβ^−^ TCRγδ^−^ CD11b^−^ CD11c^−^). **a** Graphs show compilations of intestinal ILC2 proportions (**b**) and numbers (**c**) in the siLP and cLP. **d**, **e** IL-5 and IL-13 production from siLP and cLP ILC2 (lineage-negative CD90.2^+^Sca-1^+^) from naïve WT and *Itk*^*−/−*^ mice. **f**, **g** ILC2 proportions from the bone marrow (BM), lung, and mesenteric lymph nodes (mLN) of naïve WT and *Itk*^*−/−*^ mice (**f**) along with compilations of data (**g**). **h** The proportions of various ILC subsets in the siLP and cLP were analyzed. Intestinal ILC2 and ILC3 frequencies were enumerated from the gating shown in Supplementary Fig. 1 A. Data are the compilation of 2–3 independent experiments using WT (*n* = 4–6) and *Itk*^*−/−*^ mice (*n* = 4–6). Statistical significance was analyzed using Student’s *t* tests (n.s., not significant; *p < 0.05; **p < 0.01; ***p < 0.001). Source data are provided as a Source Data file

Gene expression data indicate that *Itk* is highly expressed in intestinal ILC2, at levels comparable with those seen in T cells; furthermore, high expression of *Itk* was not shared with gut ILC1 or ILC3 (www.immgen.org). To determine whether ITK might function in ILC2, we isolated lymphocytes from small and large intestinal LP of WT and *Itk*^*−/−*^ mice and analyzed ILC2 populations. In both small and large intestine, ILC2 frequency in *Itk*^*−/−*^ mice was significantly reduced compared with WT (3–6-fold; Fig. 1a, b), and the numbers of ILC2 in both intestinal tissues were 6–7-fold lower in *Itk*^*−/−*^ mice relative to controls (Fig. 1c). Ex vivo stimulation of LP lymphocytes also demonstrated that ITK-deficient ILC2 were impaired in cytokine production (Fig. 1d, e). ILC2 deficit was restricted to the intestinal tissues, as BM, lung, and mesenteric lymph nodes (mLN) of *Itk*^*−/−*^ mice showed no reduction in ILC2 proportions compared with controls (Fig. 1f, g). Furthermore, assessment of steady-state intestinal ILC1, ILC2, and ILC3 examined together using the gating strategy outlined in Supplementary Fig. 1A indicated that intestinal ILC2 were the only subset profoundly affected ITK deficiency (Fig. 1h and Supplementary Fig. 1B). These data demonstrate that the absence of ITK specifically affects the ILC2 in the gut, but not other ILC populations, or ILC2 in other tissue sites.

### Impaired gut ILC2 expansion in virus-infected *Itk*^−/−^ mice

Pulmonary viral infections have been shown to induce substantial increases in lung ILC2 numbers^7,33,34^. To assess the responses of *Itk*^*−/−*^ ILC2 to virus, we infected WT and *Itk*^*−/−*^ mice with mouse gammaherpesvirus-68 (MHV68) by the intranasal route (10^3^ PFU). As previously described for respiratory syncytial virus (RSV) or influenza A virus (IAV) infection, intranasal MHV68 infection induced a twofold increase in lung ILC2 at D7, with a further increase at D14 compared with uninfected controls (Supplementary Fig. 2A-B vs. Fig. 1f, g). This response was not altered in the absence of ITK. We then challenged WT and *Itk*^*−/−*^ mice with MHV68 via intraperitoneal inoculation (10^6^ PFU), as MHV68 has been shown to replicate in the gastrointestinal (GI) epithelium^35,36^. As shown, ILC2 in the siLP and cLP of WT mice showed a twofold increase at D7 compared with uninfected controls; steady-state proportions of intestinal ILC2 were restored in WT mice by D14 (Supplementary Fig. 2C, D vs. Fig. 1a, b). In contrast, intestinal ILC2 in *Itk*^*−/−*^ mice remained at low levels after infection, at both D7 and D14 (Supplementary Fig. 2C-D).

We also examined other ILC subsets in the intestine after MHV68 by IP inoculation. Although there were modest decreases in ILC3 and ILC1 in cLP of *Itk*^*−/−*^ mice at D14 compared with WT, no significant differences were consistently observed in intestinal ILC3 or ILC1 in MHV68-infected WT versus *Itk*^*−/−*^ mice at both timepoints, as was seen for intestinal ILC2 (Supplementary Fig. 3). To determine whether gut ILC2 were required for effective viral control in the intestine, we challenged *Rora*^*fl/fl*^ x *Il7ra-Cre* (*Rora*^*fl/fl*^) mice or littermate controls (*Rora*^*+/+*^ and *Rora*^*+/fl*^) with IP MHV68 infection, as *Rora*^*fl/fl*^ mice are known to lack ILC2 in all tissues^16^. Despite this ILC2 deficiency, *Rora*^*fl/fl*^ mice had similar MHV68 viral genome copy numbers in both the spleen and small/large intestine at D14 compared with controls (Supplementary Fig. 4). Collectively, these data indicate that *Itk*^*−/−*^ mice have an intestinal ILC2 deficit that cannot be overcome by a lytic viral insult to the GI epithelium.

### BM *Itk*^*-/-*^ ILC2P have normal gut-homing receptor expression

**Fig. 2:**
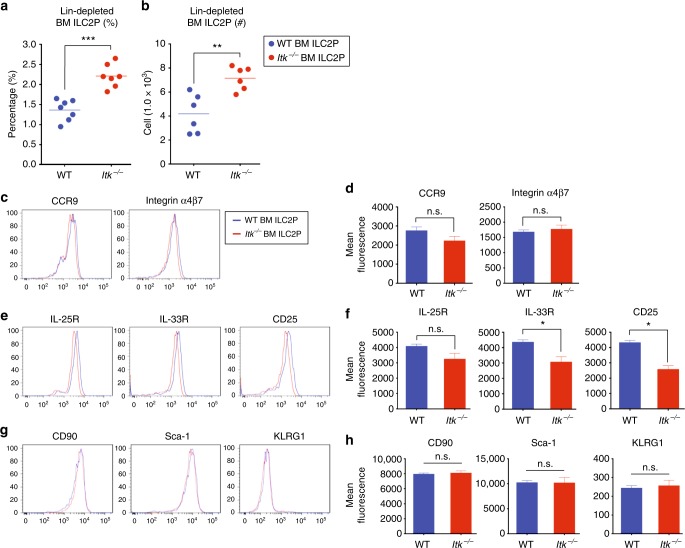
BM *Itk*^*−/−*^ ILC2P exhibit normal gut-homing receptor expression. **a**, **b** The proportions and numbers of WT and *Itk*^*−/−*^ ILC2 precursors were analyzed from lineage-depleted BM cells. **c**–**h** The expression of gut-homing receptors, CCR9 and integrin α4β7 (**c**, **d**, top panel), cytokine receptors, IL-25R, IL-33R, and CD25 (**e**, **f**, middle panel), and ILC2 markers, CD90, Sca-1, and KLRG-1 (**g**, **h**, bottom panel), on WT and *Itk*^*−/−*^ BM ILC2P were analyzed. Data are the compilation of 2–3 independent experiments using WT (*n* = 4–7) and *Itk*^*−/−*^ mice (*n* = 4–7). Bar graphs with error bars show the averages and the SEMs. Statistical significance was analyzed using Student’s *t* tests (n.s., not significant; *p < 0.05; **p < 0.01; ***p < 0.001). Source data are provided as a Source Data file

Our examination of ILC2 in the BM showed a modest increase in the proportions of these cells in *Itk*^*−/−*^ mice compared with WT (Fig. 1f). To assess whether ITK might function in ILC2 development, we performed additional characterization of BM ILC2 precursors. After depleting lineage-positive cells, we compared the frequencies and numbers of ILC2 precursors in naïve WT and *Itk*^*−/−*^ mice. Similar to our findings shown above in the absence of lineage depletion (Fig. 1f), lineage-depleted population analyses revealed modest increases in the frequency and cellularity of *Itk*^*−/−*^ BM ILC2P compared with WT (Fig. 2a, b). However, the magnitude of these changes was far smaller than the reduction in ILC2 numbers seen in the intestinal tissue of *Itk*^*−/−*^ mice (Fig. 1a–c).

Compared with other ILC subsets, ILC2 precursors in the BM have been shown to express gut-homing receptors, CCR9 and integrin α4β7, and to traffic efficiently to the intestine without requiring priming in secondary lymphoid organs^8,24,26^. Therefore, we examined gut-homing receptor expression on BM ILC2P from WT and *Itk*^*−/−*^ mice. As shown, we did not observe any significant differences in the expression of CCR9 and integrin α4β7 between *Itk*^*−/−*^ ILC2P and WT ILC2P (Fig. 2c, d). We also examined ILC2-associated cytokine receptors, IL-25R, IL-33R, and CD25 (Fig. 2e, f), and surface markers, CD90, Sca-1, and KLRG-1 (Fig. 2g, h). While we found modest reductions of IL-33R and CD25 expression on *Itk*^*−/−*^ ILC2P compared with controls, the magnitude of these changes was small. Taken together, these data overall do not reveal major differences between WT and *Itk*^*−/−*^ ILC2P in the BM. Furthermore, the data indicate that the ITK deficiency is not likely to affect the gut-homing potential of ILC2 generated in the BM, at least not due to altered expression of gut-homing receptors.

### ITK is dispensable for gut-homing receptors on gut ILC2

**Fig. 3:**
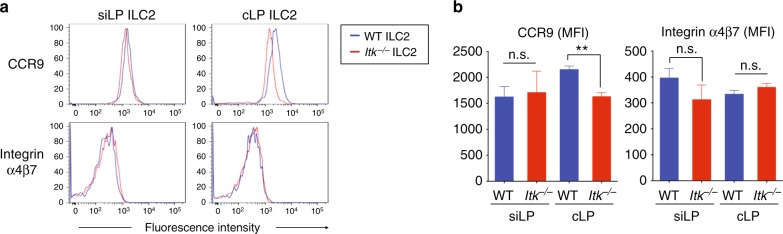
ITK is not required for ILC2 gut-homing receptor. **a**, **b** The expression of CCR9 and integrin α4β7 on siLP or cLP ILC2 were analyzed (**a**). Compilation MFI data from three independent experiments are shown (**b**). Data are the compilation of three independent experiments using WT (*n* = 5) and *Itk*^*−/−*^ mice (*n* = 5). Statistical significance was analyzed using Student’s *t* tests (n.s., not significant; **p < 0.01). Source data are provided as a Source Data file

To investigate further a potential role for ITK in gut-homing receptor expression on ILC2, we assessed CCR9 and integrin α4β7 levels on ILC2 in the small and large intestinal LP from naïve WT and *Itk*^*−/−*^ mice. In spite of being present at reduced cellularity, the expression levels of homing receptors on *Itk*^*−/−*^ ILC2 from the small intestinal LP were similar to those on WT ILC2 from this site (Fig. 3a, b, left panel). In the colonic LP, CCR9 expression on *Itk*^*−/−*^ ILC2 shown was modestly reduced compared with that on WT cLP ILC2, whereas integrin α4β7 expression levels were similar (Fig. 3a, b, right panel).

As previously reported, retinoic acid (RA) is neither required for the expression of gut-homing receptors nor for the tissue homing ability of ILC2 to the intestine^24^. Nonetheless, we investigated the effects of TGF-β and/or RA on ILC2 gut-homing receptor expression in the absence of ITK. For this analysis, we isolated lineage-depleted WT and *Itk*^*−/−*^ BM cells and cultured them in vitro with IL-2, IL-7, and IL-33 in presence of RA and/or TGF-β for 5 days. As shown, and consistent with previously reported data^24^, we neither observed any detectable differences in integrin α4, integrin β7, or CCR9 expression on cultured BM ILC2P in response to RA alone, nor did we observe differences between WT and *Itk*^*−/−*^ ILC2P (Supplementary Fig. 5A and 5D–F). TGF-β alone had divergent effects on integrin α4 and integrin β7 in both WT and *Itk*^*−/−*^ ILC2P (Supplementary Fig. 5B, D and E). In combination, RA plus TGF-β induced both CCR9 and integrin α4β7 on BM ILC2P, with only modest differences seen by ILC2P from WT versus *Itk*^*−/−*^ mice (Supplementary Fig. 5C and 5D–F). Taken together, these data indicate that ITK is not strongly regulating the expression levels of gut-homing receptors on ILC2 in response to RA and TGF-β stimulation.

### *Itk*^*−/*−^ ILC2 show a modest defect in response to IL-33

**Fig. 4:**
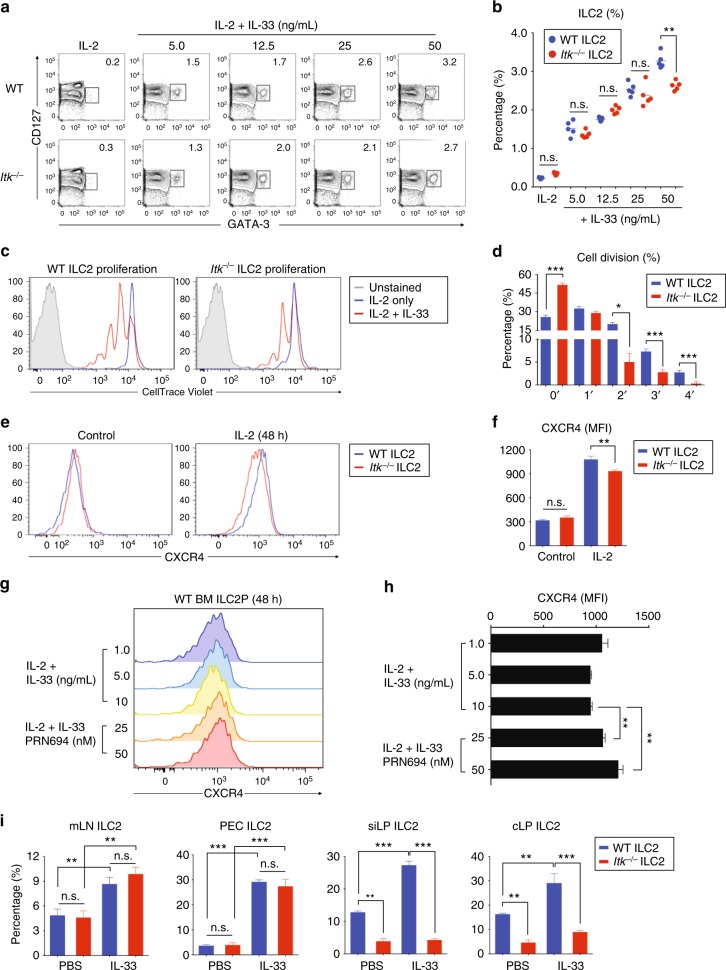
*Itk*^*−/−*^ ILC2 responses to IL-33 are modestly impaired. **a**, **b** Lineage-depleted WT and *Itk*^*−/−*^ BM cells were cultured with IL-2 plus IL-33 (5.0 to 50 ng/mL) for 2 days and analyzed for ILC2 precursors (**a**). Compiled data from two independent experiments are shown (**b**). **c**, **d** Lineage-depleted WT and *Itk*^*−/−*^ BM cells were labeled with CellTrace Violet and assessed for IL-33-induced proliferation after 2 days culture (**c**). Compiled percentage of cells in each cell division peak from two individual experiments is shown (**d**). **e**–**h** CXCR4 expression on lineage-depleted BM WT and *Itk*^*-−/−*^ ILC2 precursors was analyzed in the presence or absence of IL-2 after 2 days culture (**e**). Compiled MFIs from three independent experiments are shown (**f**). CXCR4 expression on cultured BM WT ILC2P was analyzed in the presence or absence of PRN694 after culture with IL-2 and IL-33 for 2 days (**g**). Calculated MFIs from the compilation of two experiments are shown (**h**). **i** Mice were injected daily with IL-33 for 7 days, and the percentages of ILC2 from mesenteric lymph nodes, peritoneal cavity exudates, and the small and large intestinal lamina propria were assessed. Data are the compilation of two independent experiments using WT (*n* = 4-5) and *Itk*^*-/-*^ mice (*n* = 4-5). Bar graphs with error bars show the averages and the SEMs. Statistical significance was analyzed using Student’s *t* tests (n.s., not significant; *p < 0.05; **p < 0.01; ***p < 0.001). Source data are provided as a Source Data file

Based on our findings that *Itk*^*−/−*^ ILC2 showed modest reductions in IL-33R expression, we considered whether these cells might have impaired responses to IL-33. To determine this, we cultured lineage-depleted BM cells with IL-2 plus increasing doses of IL-33 (5.0–50 ng/mL) for 2 days and compared the expansion of ILC2 in the populations of WT and *Itk*^*−/−*^ BM cells. In the presence of IL-2 plus IL-33, both WT and *Itk*^*−/−*^ BM cells showed a comparable ILC2 induction in response to comparatively low IL-33 concentrations (5.0–25 ng/mL); however, at the highest dose of IL-33 (50 ng/mL), we observed a modest reduction in the proportions of ILC2 in *Itk*^*−/−*^ BM versus control BM cultures (Fig. 4a, b). Next, we assessed the proliferation of BM-derived ILC2P in response to IL-33. We labeled lineage-depleted BM cells with CellTrace Violet and compared proliferation in response to IL-33 after 2 days in the presence of IL-2 ± IL-33. IL-33 induced substantial cell proliferation of WT BM cells. In contrast, ITK-deficient BM cells underwent significantly less proliferation (Fig. 4c). Analyses of the percentages of cells in each division peak confirmed that *Itk*^*−/−*^ BM cultures had increased proportions of undivided cells and had reduced percentages of cells in peaks representing 2–4 cell divisions compared with controls (Fig. 4d).

As we observed modest increases in the numbers of *Itk*^*−/−*^ ILC2P in the BM compared with controls (Fig. 2b) and reduced expression of IL-33R on these cells (Fig. 2e and f), we considered whether ITK might regulate BM egress of ILC2P via expression of CXCR4. CXCR4 has been found to promote tissue retention of lymphocytes in the BM^37^. Therefore, we compared CXCR4 expression of untreated BM ILC2P directly ex vivo from naïve WT and *Itk*^*−/−*^ mice and also examined CXCR4 expression after culture of lineage-depleted BM cells with IL-2 for 48 h. We found no differences in CXCR4 expression on *Itk*^*−/−*^ versus WT BM ILC2P directly ex vivo, and a slight reduction on *Itk*^*−/−*^ BM ILC2P after culture in IL-2 (Fig. 4e, f). We then assessed whether *Itk*^*−/−*^ ILC2P showed impaired CXCR4 downregulation after culture in IL-33, as this response was reported as a mechanism controlling ILC2P egress from the BM^25^. To test this hypothesis, we cultured lineage-depleted WT BM cells with IL-2 plus varying doses of IL-33 (1.0–10 ng/mL) in the presence or absence of PRN694 (25 or 50 nM), an ITK small molecule inhibitor. After 2 days, cells were examined for CXCR4 expression. In our hands, IL-33 did not promote CXCR4 downregulation on ILC2P in cultured lineage-depleted BM cells; however, ITK inhibition did promote a modest upregulation of CXCR4 in the presence of a high dose of IL-33 (10 ng/mL) (Fig. 4g, h). Taken together, these data indicate that the absence or inhibition of ITK had a modest effect on IL-33-induced ILC2 proliferation and only very subtle effects on CXCR4 expression.

Last, we tested the efficacy of IL-33-driven ILC2 induction in vivo by injecting IL-33 into WT and *Itk*^*−/−*^ mice, and then assessing the frequencies of ILC2 in various organs. As expected, IL-33 injection increased the ILC2 frequencies in mLN and peritoneal cavities of WT mice, with a similar magnitude of change seen in *Itk*^*−/−*^ mice (Fig. 4i). These results indicated that *Itk*^*−/−*^ ILC2 had no intrinsic defect in responding to IL-33 in vivo. Interestingly, however, IL-33 injection failed to induce intestinal ILC2 increases in siLP and cLP of *Itk*^*−/−*^ mice, whereas WT mice showed clear increases in ILC2 in these tissues (Fig. 4i). These data indicate IL-33 injection in vivo is not sufficient to induce an effective ILC2 population increase in the intestines of *Itk*^*−/−*^ mice.

### ITK has a cell-intrinsic function in gut ILC2 homeostasis

**Fig. 5:**
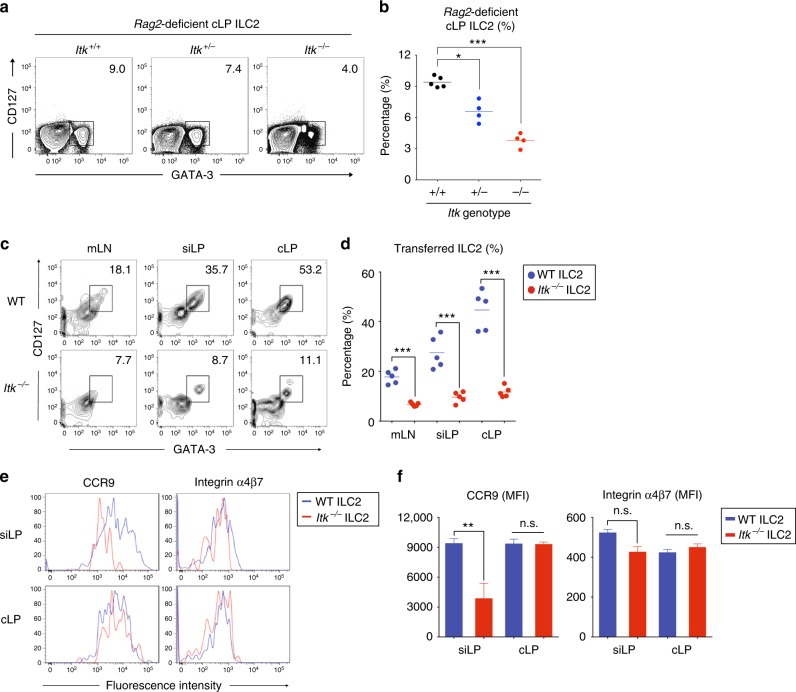
*Itk*^−/−^ ILC2 are impaired in gut tissue homeostasis. **a**, **b** The frequency of colon LP ILC2 from the *Rag2*-deficient *Itk*^*+/+*^ (WT), *Itk*^*+/*−^ (Het), and *Itk*^*−/−*^ (KO) littermates are shown (**a**). Compiled data from four to five mice per genotype are shown (**b**). **c**–**f** Lineage-depleted BM cells were cultured with IL-2, IL-7, and IL-33 in the presence of RA plus TGF-β for 3 days and then enriched by using CD25 MicroBeads. Cultured and enriched cells were adoptively transferred to *Rag1*^*−/−*^ *Il2rg*^*−/−*^ hosts, and the proportions of ILC2 in the mLN and small or large intestinal LP were examined at D3 post transfer (**c**). Compilation of data from five mice per genotype (**d**). The expression of CCR9 and integrin α4β7 on transferred WT and *Itk*^*−/−*^ ILC2 in the small intestinal LP was examined (**e**). Compiled MFIs are shown (**f**). Data are the compilation of two independent experiments using WT (*n* = 5), *Itk*^*−/−*^ (*n* = 5), *Rag2*^*−/−*^ *Itk*^*+/+*^ (*n* = 5), *Rag2*^*−/−*^ *Itk*^*+/*−^ (*n* = 4), and *Rag2*^*−/−*^ *Itk*^*−/−*^ (*n* = 4). Bar graphs with error bars show the averages and the SEMs. Statistical significance was analyzed using Student’s *t* tests (n.s., not significant; *p < 0.05; ***p < 0.001). Source data are provided as a Source Data file

Our overall findings with BM ILC2 precursors indicated that the absence of ITK was not profoundly affecting ILC2 maturation. Nonetheless, *Itk*^*−/−*^ mice consistently exhibited a defect in intestinal ILC2 that could not be restored by in vivo administration of IL-33. We considered whether this gut-specific defect in ILC2 might result from an altered environment in the intestines of *Itk*^*−/−*^ mice caused by dysregulation of *Itk*^*−/−*^ T cells, a possibility raised by previous studies indicating cross-talk between T cells and ILC in the intestine^16,17^. First, we generated mixed WT:*Itk*^*−/−*^ BM chimeras in lethally irradiated hosts. The goal of this experiment was to provide a WT environment, including the presence of WT intestinal T cells, in which to assess the development and homeostasis of ILC2 arising from *Itk*^*−/−*^ BM precursors. However, as shown in Supplementary Fig. 6, we failed to recover adequate numbers of cells from the recipients due to lethality after BM transplantation. As an alternative, we crossed *Itk*^*−/−*^ mice to *Rag2*^*−/−*^ mice, and assessed ILC2 frequencies in the intestine in the absence of T cells. As shown in Fig. 5a and b, ILC2 frequencies in the cLP of *Rag2*^*−/−*^ *Itk*^*+/−*^ or *Rag2*^*−/−*^ *Itk*^*−/−*^ littermates were reduced compared with those seen in cLP of *Rag2*^*−/−*^ *Itk*^*+/+*^ littermates. These results support an ILC2-intrinsic role for ITK in ILC2 homeostasis in the intestine that is independent of the presence of T cells. In a second experiment, we addressed whether the absence of ITK altered the migration and/or survival of ILC2 in the intestine after adoptive transfer. To examine this, we cultured lineage-depleted WT and *Itk*^*−/−*^ BM cells with IL-2, IL-7, and IL-33 in the presence of RA plus TGF-β, to fully induce gut-homing receptors in vitro. After culture for 3 days, we enriched cultured cells with CD25 MicroBeads to further isolate ILC2, and then adoptively transferred these cells into *Rag1*^*−/−*^ *Il2rg*^*−/−*^ hosts, which have neither adaptive immune cells nor innate lymphocytes. At day 3 post transfer, we isolated lymphocytes from mLN, siLP, and cLP to examine the frequencies of transferred ILC2. We observed that WT ILC2 could be efficiently recovered from the gut-draining LN and LP of both the small and large intestine, and were abundantly represented in cLP tissue (Fig. 5c, d). In contrast, *Itk*^*−/−*^ ILC2 numbers were greatly reduced in all intestinal tissues compared with WT ILC2 (Fig. 5c, d). We also analyzed the expression levels of CCR9 and integrin α4β7 on transferred WT and *Itk*^*−/−*^ ILC2 in siLP and cLP. Although CCR9 expression was reduced on siLP of *Itk*^*−/−*^ ILC2, no other differences between WT and *Itk*^*−/−*^ transferred ILC2 were observed (Fig. 5e, f). ILC2 isolated from the spleen and mLN of recipients also failed to reveal prominent differences in gut-homing receptor expression between transferred WT and ITK-deficient cells (Supplementary Fig. 7). Taken together, these data show that a deficiency in ITK leads to a cell-intrinsic defect in ILC2 recovery from the gut tissue, but suggest that the underlying cause of this defect is unlikely to be due to impaired gut-homing receptor expression.

### ITK deficiency affects intestinal tissue integrity

**Fig. 6:**
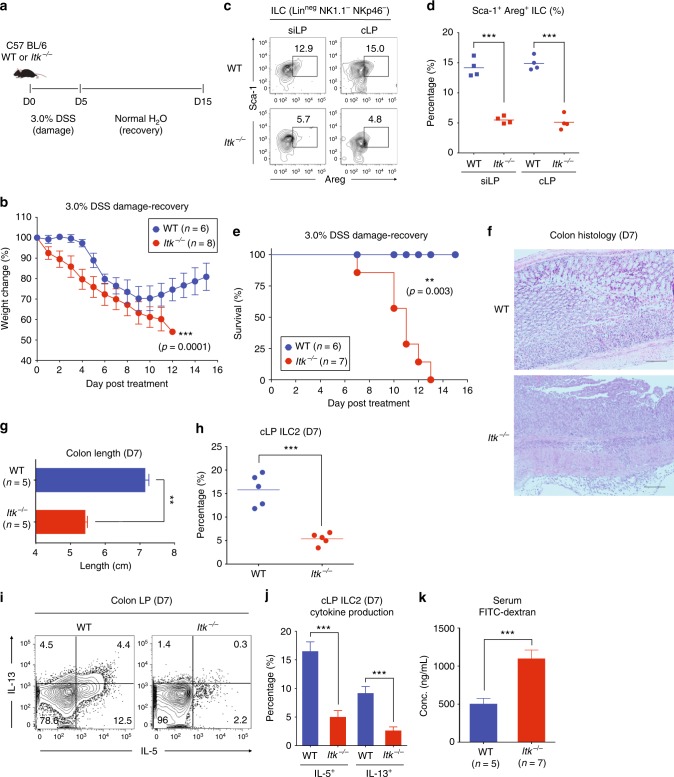
*Itk*^−/−^ mice have impaired intestinal tissue integrity. **a**, **b** In total, 3.0% DSS-dissolved water was given to WT and *Itk*^*−/−*^ mice for 5 days and then changed to normal drinking water for 10 days (**a**). Weight changes of treated mice were monitored daily (**b**). **c**, **d** The proportion of amphiregulin-producing Sca-1^+^ ILC (lineage-negative NK1.1^−^ NKP46^−^) from siLP and cLP tissue from WT and *Itk*^*−/−*^ mice in steady-state conditions. **e** The survival of 3.0% DSS-treated WT and *Itk*^*−/−*^ mice from D0 to D15 is shown. **f**–**j** WT and *Itk*^*−/−*^ mice were treated with 3.0% DSS water for 7 days and histology of distal colon was examined (**f**). The length of colons from each group was measured (**g**) and colon ILC2 proportions were examined at D7 (**h**). Isolated lymphocytes from colon LP of WT and *Itk*^*−/−*^ mice at D7 were stimulated with PMA and ionomycin for 5 h and stained for IL-5 and IL-13. Data show cells gated on lineage-negative CD90^+^ Sca-1^+^ cells (**i**). Complied data are shown (**j**). **k** Intestinal permeability of WT and *Itk*^*−/−*^ mice at steady-state was measured by assessing serum levels of FITC-dextran 4 h after oral administration. Data are the compilation of two independent experiments using WT (*n* = 4-6) and *Itk*^*−/−*^ mice (*n* = 4-8). Bar graphs with error bars show the averages and the SEMs. Statistical significance was analyzed using Student’s *t* tests (**p < 0.01; ***p < 0.001). Source data are provided as a Source Data file

**Fig. 7:**
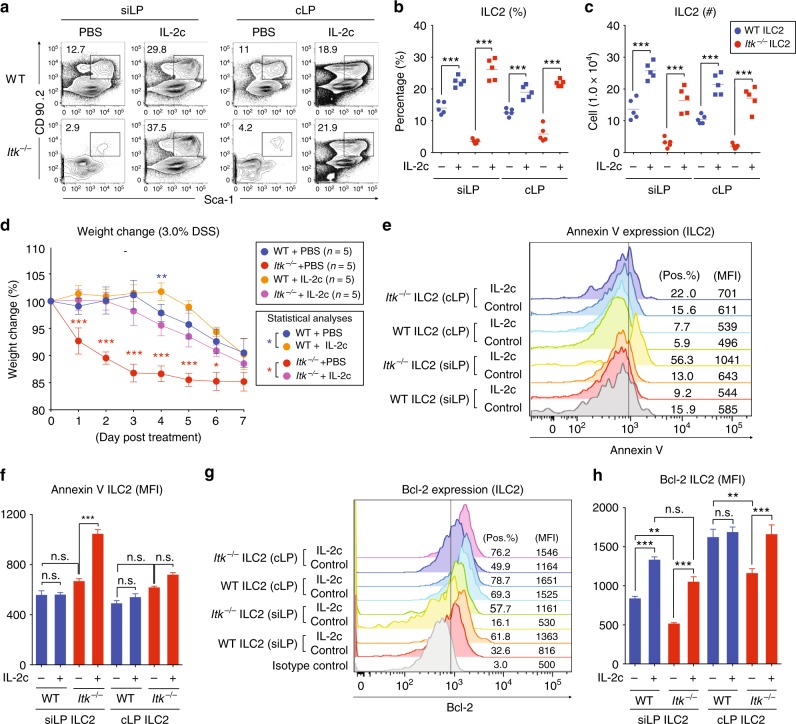
IL-2 complex injection restores gut ILC2. **a**–**c** PBS or IL-2 and anti-IL-2 mAb complexes (IL-2c) were injected daily IP for 4 days, and the proportions of gut ILC2 (CD90^+^ Sca-1^+^) from the lamina propria of small and large intestines were examined after gating on lineage-negative cells (**a**). Compiled gut ILC2 proportions (**b**) and numbers (**c**) from two independent experiments is shown. **d** PBS- or IL-2c-treated WT and *Itk*^*−/−*^ mice were administered 3.0% DSS for 7 days, and weight was monitored daily. Statistical analyses between PBS-treated versus IL-2c-treated WT mice (blue asterisk) and PBS-treated versus IL-2c-treated *Itk*^*−/−*^ mice (red asterisk) are shown. **e**–**h** The expression of Annexin V (**e**) and Bcl-2 (**g**) in siLP and cLP ILC2 from PBS- or IL-2c-treated WT and *Itk*^*−/−*^ mice was examined. Compiled MFIs from two independent experiments are shown (**f** and **h**). Data are the compilation of two independent experiments using WT (*n* = 4–5) and *Itk*^*−/−*^ mice (*n* = 4–5). Bar graphs with error bars show the averages and the SEMs. Statistical significance was analyzed using Student’s *t* tests (n.s., not significant; *p < 0.05; **p < 0.01; ***p < 0.001). Source data are provided as a Source Data file

Our results thus far indicated that the absence of ITK caused an intestinal ILC2 defect. Given the reparative function of intestinal ILC2 in helminthic infection or autoimmune disease conditions^11–13,38^, we considered whether the intestinal ILC2 defect in *Itk*^*−/−*^ mice affected GI tissue integrity and repair from intestinal damage. To test this, we challenged WT and *Itk*^*−/−*^ mice with 3.0% DSS-dissolved water for 5 days to induce intestinal damage, and then restored normal drinking water for an additional 10 days to monitor intestinal tissue repair (Fig. 6a). As shown in Fig. 7b, DSS-treated WT mice exhibited weight loss beginning at day 5 post treatment and then recovered and gradually regained lost weight starting at day 9 (Fig. 6b). In contrast, *Itk*^*−/−*^ mice displayed an immediate weight loss beginning on day 1, and showed a continuous weight loss during the recovery period (D6-D15) (Fig. 6b), consistent with impaired amphiregulin production by *Itk*^*−/−*^ intestinal ILC2 under steady-state conditions (Fig. 6c, d). Failure to recover from the DSS-induced damage was ultimately fatal in *Itk*^*−/−*^ mice (*n* = 7, mean survival = 11.0 days), while DSS-treated WT mice showed no lethality (*n* = 6) (Fig. 6e). Consistent with these data, histological analysis of DSS-treated *Itk*^*−/−*^ colons at D7 post treatment revealed more cellular infiltrates than seen in WT colon histology at D7 (Fig. 6f), and the length of *Itk*^*−/−*^ colons (*n* = 5) was shorter compared with WT colons (*n* = 5), indicating more severe inflammation in the colons of *Itk*^*−/−*^ mice (Fig. 6g). As expected, the frequencies of ILC2 in *Itk*^*−/−*^ colonic LP were greatly reduced in DSS-treated *Itk*^*−/−*^ mice compared with controls, as we observed under steady-state conditions (Fig. 6h). The role of ILC2 in the maintenance of tissue integrity is mediated by effector cytokines produced by these cells, such as IL-5 and IL-13, which induce tissue repair, mucus production, and goblet cell hyperplasia^39^. Therefore, we performed intracellular cytokine staining for IL-5 and IL-13 with isolated colonic LP lymphocytes from DSS-treated WT and *Itk*^*−/−*^ mice at D7 post treatment. As shown in Fig. 6i and j, IL-5 and IL-13 production from cLP ILC2 in *Itk*^*−/−*^ mice was significantly reduced in comparison with the production from WT cLP ILC2.

To independently test whether *Itk*^*−/−*^ mice had impaired intestinal tissue integrity, we administered FITC-dextran to untreated WT and *Itk*^*−/−*^ mice via oral gavage, and compared the levels of serum FITC-dextran after 4 h as an indicator of intestinal permeability. Consistent with our findings in the DSS-induced colitis model, we observed a higher level of FITC-dextran in the serum collected from *Itk*^*−/−*^ mice than WT mice (Fig. 6k). These data strongly suggest that ITK deficiency, associated with the ILC2 defect in the colon, leads to impaired intestinal integrity under steady-state conditions, as well as in response to intestinal damage.

### IL-2 complex injection restores gut ILC2 in *Itk*^−/−^ mice

Our results outlined above indicated a defect in gut-homing migration of ILC2 in *Itk*^*−/−*^ mice. We considered whether this defect might be due to impaired ILC2 homeostasis in the gut, as a component of the failure to observe high numbers of *Itk*^*−/−*^ ILC2 in the intestinal tissues of *Rag*^*−/−*^*Il2rg*^*−/−*^ mice after adoptive transfer. Specifically, we considered whether the ITK deficiency might contribute to a defect in ILC2 cell survival in the gut as a result of a reduced IL-2 production by *Itk*^*−/−*^ T cells^19,40^. To test this hypothesis, we mixed IL-2 and anti-IL-2 mAb (JES6-1) to make IL-2 complexes (IL-2c), and injected these complexes daily into WT and *Itk*^*−/−*^ mice. After 4 days, we analyzed ILC2 in the intestines. As reported previously^19,20,40^, IL-2c injection into WT mice increased the frequencies of ILC2 (CD90^+^ Sca-1^+^) in siLP and cLP compared with PBS-injected control mice (Fig. 7a, b). Surprisingly, IL-2c injection into *Itk*^*−/−*^ mice showed a profound effect on ILC2, leading to complete restoration of ILC2 proportions and numbers in the siLP and cLP, to a level similar to that seen in IL-2c-treated WT mice (Fig. 7a, c). In contrast, injection of these IL-2c had only modest effects on the numbers of FoxP3^+^ CD4^+^ T cells (Treg), and no effect on the numbers of conventional (FoxP3^−^) CD4^+^ T cells in the intestinal tissue of either WT or *Itk*^*−/−*^ mice (Supplementary Fig. 8A–D).

To determine whether restoration of ILC2 in the intestine of *Itk*^*−/−*^ mice by IL-2c improved the responses of these mice to intestinal tissue damage, we treated mice with DSS (Fig. 7d). Although IL-2c treatment provided a marginal improvement in weight loss after DSS-induced damage in WT mice, IL-2c-treated *Itk*^*−/−*^ mice were markedly improved compared with controls (DSS but no IL-2c; Fig. 7d). In fact, IL-2c treatment of *Itk*^*−/−*^ mice restored their ability to respond to intestinal tissue damage to that of control-treated WT mice.

These findings suggested that *Itk*^*−/−*^ intestinal ILC2 might have impaired survival potential, leading to their reduced numbers and inability to expand in numbers following virus-induced or chemical-induced intestinal tissue damage. To assess this, we first examined Annexin V staining on siLP and cLP ILC2, before and after IL-2c treatment. After IL-2c treatment, we observed increased Annexin V on *Itk*^*−/−*^ ILC2, while all other populations were similar (Fig. 7e, f). These data indicate that the increased numbers of ILC2 in IL-2c-treated *Itk*^*−/−*^ mice are undergoing a higher rate of apoptosis compared with all other ILC2 populations. As IL-2 stimulation of T cells is known to upregulate Bcl-2^41,42^, we assessed Bcl-2 levels in intestinal ILC2, before and after IL-2c injection. We first observed that *Itk*^*−/−*^ intestinal ILC2 in both the siLP and the cLP had markedly reduced Bcl-2 levels under steady-state conditions (Fig. 7g, h). In response to IL-2c, Bcl-2 levels in *Itk*^*−/−*^ ILC2 increased substantially, and achieved levels comparable with those seen in the respective WT ILC2 populations. Collectively, these data support the conclusion that the defect in intestinal ILC2 in *Itk*^*−/−*^ mice results from impaired ILC2 survival in the intestinal environment.

## Discussion

In this study, we show that ITK deficiency specifically affects ILC2 homeostasis in the intestine, but not of ILC2 residing in other tissues, or of other innate ILC subsets, such as ILC1 and ILC3. As reported previously, TCR signaling components and cytokine receptors are known to be upregulated in ILC precursors during their early development upon the expression of T-cell lineage transcription factors, e.g., TCF-1, GATA-3, and Bcl11b^22,23,26–28^. However, the role of these genes in mature ILC positioned in the peripheral tissues has not been well characterized. Given CD25 expression among ILCs and the responsiveness to IL-2^3,5,20,40^, the function of ITK in ILC2 might be involved in IL-2-associated ILC2 homeostasis in the periphery. In CD4^+^ T cells, ITK is required for T_H_2-mediated responses, which are dependent on IL-2/STAT5-mediated signaling^43,44^. Supporting this notion, STAT5 deficiency affects GATA-3 expression in intestinal ILC2 while RORγt expression in ILC3 is largely normal^45^.

In response to viral infections, such as RSV, IAV, or rhinovirus, lung-resident ILC2 are activated by tissue damage via the production or release of IL-25 or IL-33^6,18,33,46–48^. However, no studies have examined potential changes in intestinal ILC2 following virus infection. Considering the increase in IL-25-producing tuft cells after murine norovirus infection^49^, intestinal ILC2 numbers would be likely to increase, as is seen following intestinal parasite infections^50,51^. The role of intestinal ILC2 during pathogen infection is to promote intestinal epithelium integrity via IL-5, IL-13, and amphiregulin, which leads goblet hyperplasia and mucus production^11–13,18^. Consistent with these findings *Itk*^*−/−*^ mice, having few ILC2 in the intestine, displayed more severe responses to DSS-induced intestinal tissue damage compared with WT mice; further, intestinal tissue permeability of *Itk*^*−/−*^ mice was greatly increased under steady-state conditions. Although the severe response to DSS-induced damage could be affected by a potential defect in T cells in *Itk*^*−/−*^ mice, DSS colitis is considered to be largely dependent on innate immunity^52^. The onset of weight loss in *Itk*^*−/−*^ mice to DSS treatment is immediate (D1 of post treatment), which argues against a contribution of T cells to disease progression in DSS-treated *Itk*^*−/−*^ mice.

In the present study, we showed IL-2c injection restores intestinal ILC2 numbers in *Itk*^*−/−*^ mice, while IL-33 injection failed to exert such an effect. As we observed comparable proliferation of both WT and *Itk*^*−/−*^ ILC2 to IL-33 in other organs in vivo, it is possible that the initial number of gut ILC2 in *Itk*^*−/−*^ mice was too low to show robust expansion in the intestinal LP. However, *Itk*^*−/−*^ ILC2 in the intestine did exhibit extensive expansion following IL-2c injection, with a fold expansion even higher than seen for WT intestinal ILC2. Previous reports showed that IL-2, a cytokine predominantly produced by CD4^+^ T cells, promotes the proliferation, expansion, and also effector function of ILC2, suggesting a reciprocal interaction between CD4^+^ T cells and ILC2^16,19,40,53^. This notion also correlates with our findings of reduced CD4^+^ T-cell numbers in the intestinal mucosa, but not the lungs, of *Itk*^*−/−*^ mice under steady-state conditions (Supplementary Fig. 8). Furthermore, as we observed comparable gut-homing receptor expression on both WT and *Itk*^*−/−*^ ILC2, the intestinal ILC2 defect in *Itk*^*−/−*^ mice is unlikely to be accounted for by a cell-intrinsic migration defect, despite the reduction in *Itk*^*−/−*^ ILC2P seen 3 days after transfer of ILC2 precursors into *Rag1*^*−/−*^ *Il2rg*^*−/−*^ hosts.

Administration of IL-2/anti-IL-2 antibody complexes (JES6-1A12) restored the population of intestinal ILC2 in *Itk*^*−/−*^ mice. Based on this finding, it is plausible that CD4^+^ T cells are important in the tissue maintenance of ILC2 in the intestine via IL-2 production. In these studies, we did not detect alterations in the populations of conventional CD4^+^ or CD8^+^ T cells in the intestinal tissue due to the preferential binding of the JES6-1A12/IL-2 complexes to Treg over conventional T cells^54–56^. Future studies are warranted to test whether adoptive transfer of CD4^+^ T cells or injection of IL-2 complexes formed with the S4B6 clone of anti-IL-2 antibody into *Itk*^*−/−*^ mice would lead to the recovery of ILC2 in the gut tissues of these mice.

Gomez-Rodriguez et al. recently reported late defects of the lung ILC2 in *Itk*^*−/−*^ mice upon papain challenge, and their results suggest that impaired T-cell function and IL-2 production may result in these defects^57^. Despite a comparable lung ILC2 population in WT and *Itk*^*−/−*^ mice after intranasal MHV68 challenge in our studies, our data on intestinal ILC2 support the notion that reduced T-cell numbers are critical for ILC2 homeostasis in the tissue. Gomez-Rodriguez et al. also reported that IL-2 or constitutively active STAT5 restored the impaired effector function of *Itk*^*−/−*^ T_H_9 cells, a subset of effector CD4^+^ T cells with functional similarity to ILC2^57^. These data strengthen our conclusion that a cell-intrinsic defect in *Itk*^*−/−*^ ILC2 is due to impaired homeostasis and survival, possibly related to insufficient IL-2.

Our data indicate that IL-2 promotes the survival and persistence of intestinal ILC2. One likely mechanism is IL-2-induced activation of STAT5 leading to transcription of the STAT5 target gene Bcl-2^45,58^. Although IL-15 can also affect cell survival of ILC via STAT5, IL-15-mediated signaling is not required for the homeostasis of ILC2^45^. Of note, CD122 expression levels in ILC2 are lower than those in ILC1 and ILC3, indicating that STAT5 signaling in ILC2 would likely be more dependent on IL-2^59^. Interestingly, Villarino et al. suggested that this loss of intestinal ILC2 in *Stat5*-deficient mice is due to defects in later development, i.e., tissue homing or peripheral homeostasis, not due to defects in ILC2 progenitors^45^. These data align with our conclusions, and support the notion that ITK is required in ILC2 for optimal responses to IL-2/STAT5 signaling. Consistent with this, *Itk*^*−/−*^ ILC2 displayed reduced Bcl-2 expression even in response to in vivo IL-2 stimulation, similar to that observed in *Stat5*-deficient ILC2^45^. Interestingly, we did not detect increased proportions of apoptotic ILC2 in *Itk*^*−/−*^ intestines compared with WT under steady-state conditions. We speculate that these apoptotic cells are rapidly cleared from the tissue by resident macrophages or other phagocytic cells. Taken all together, our data argue that the ITK-associated defect in intestinal ILC2 is due to IL-2 availability in the microenvironment along with impaired cell survival due to poor expression of Bcl-2.

Overall, we show that ITK regulates ILC2 tissue homeostasis in the intestine, and consequently, is required to promote intestinal epithelium barrier integrity. These findings suggest that a deficiency in ITK may affect the severity of enteric pathogen infection or inflammatory bowel disease via impaired ILC2-mediated tissue homeostasis.

## Methods

### Mice

C57BL/6 WT and congenic CD45.1 mice were purchased from Taconic Biosciences. All purchased mice were maintained with *Itk*^*−/−*^ in our vivarium for several generations. C57BL/6 *Rag1*^*−/−*^ *Il2rg*^*−/−*^ mice were provided by Michael A. Brehm (UMMS). C57BL/6 *Il7ra-Cre* x *Rora*^*fl/fl*^ were bred and maintained in the animal facility of the Skirball Institute at NYU. Mice were housed in SPF conditions in accordance with UMMS or NYU IACUC guidelines.

### Antibodies for flow cytometric analysis

Cells were stained with anti-mouse CD3 (145-2C11), CD11b (M1/70), CD11c (N418), CD19 (6D5), CD25 (PC61.5), CD45.1 (A20), CD45.2 (104), CD90.2 (53-2.1), CD127 (A7R34), CD199 (eBioCW-1.2), CD335 (29A1.4), IL-25R (MUNC33), integrin α4 (9F10), integrin β7 (FIB504), integrin α4β7 (DATK32), KLRG-1 (2F1), ST2 (RMST2-33), NK1.1 (PK136), Sca-1 (D7), TCRβ (H57-597), TCRγδ (eBioGL3), CXCR4 (L276F12), GATA-3 (L50-823), RORγt (AFKJS-9), T-bet (4B10), IL-5 (TRFK5), IL-13 (eBio13A), amphiregulin (206220), and anti-rat IgG (BD Biosciences, BioLegend, R&D, and Invitrogen).

### MHV68 DNA copy number measurement via q-PCR

Tissue DNA samples were subjected to q-PCR specific for viral gene ORF75c, and values were compared with a standard curve generated using a plasmid containing ORF75c. PCR primers were 5′-AAATGGTGAAAGCCATTTTGA-3′ (forward) and 5′-CCACCATCGCATAACAGTTG-3′ (reverse).

### Lineage-depleted BM cell isolation and cell cultures

BM cells were counted and Lin^-^ BM cells were isolated with mouse Lineage Cell Depletion Kit (Miltenyi Biotec). For cell proliferation assays, Lin^−^ BM cells were labeled with CellTrace Violet Cell Proliferation Kit (Invitrogen) and cultured in the presence of IL-2 (20 ng/mL, Invitrogen) ± IL-33 (50 ng/mL, R&D Systems) for 2 days. For gut-homing receptor induction, Lin^−^ BM cells were cultured with IL-2 (20 ng/mL), IL-7 (20 ng/mL, Invitrogen), IL-33 (20 ng/mL) in the presence or absence of RA (1.0 nM, Sigma-Aldrich) and/or TGF-β (5.0 ng/mL, R&D Systems) for 5 days. For measuring CXCR4 expression in response to IL-33, Lin^−^ BM cells were cultured with IL-2 (10 ng/mL) ± IL-33 (1 to 10 ng/mL) plus or minus ITK inhibitor PRN694^60^ (Principia Biopharma) for 3 days.

### In vivo injection of IL-33 or IL-2 complexes

Mice received daily IP injections of PBS or IL-33 (0.3 μg) seven times. For IL-2 complexes, mouse IL-2 (1.0 μg) and anti-IL-2 (5.0 μg) (JES6-1A12, Invitrogen) were mixed and incubated at 37 ^°^C for 30 min. Incubated IL-2 complex was given IP daily for 4 days.

### Generation of BM chimeras

Harvested BM cells from WT (CD45.1 or GFP reporter) and *Itk*^*−/−*^ mice (CD45.2) were mixed in 1:1, 1:3, and 1:5 ratios. The WT BM recipients were irradiated with 550 rad twice with a 4 h interval between doses. A total of 2.0 × 10^7^ BM cells were intravenously injected.

### Adoptive transfer of BM ILC2P

Lin^−^ BM cells were cultured with IL-2, IL-7, and IL-33 plus RA and TGF-β for 3 days and then enriched with mouse CD25 MicroBead Kit. CD25-enriched Lin^−^ BM cells (1.0 × 10^6^ cells) were intravenously injected to naïve Rag1^*−/−*^ *Il2rg*^*−/−*^ mice. Lymphocytes from mLN and intestinal tissues were isolated at D3.

### Dextran sodium sulfate-induced colitis

In total, 3.0% of DSS (MP Biomedicals) was dissolved in the drinking water, and mice were treated for 5 days and then changed to normal water for 10 days. Weight change was monitored daily, and in some experiments, mice were killed at D7.

### Measurement of intestinal permeability

Food and water were withdrawn for 6 h and mice were orally administered FITC-dextran (Sigma-Aldrich) diluted in PBS (60 mg/100 g). Serum was collected after 4 h and FITC-dextran level was measured with a UV spectrophotometer.

### Statistical analysis

All statistical analyses were performed using Prism 7 GraphPad Software. Differences between individual groups were analyzed for statistical significance using Student’s *t* tests (*p < 0.05; **p < 0.01; ***p < 0.001).

## Supplementary information

Supplementary Information

Supplementary Figure 1

Supplementary Figure 2

Supplementary Figure 3

Supplementary Figure 4

Supplementary Figure 5

Supplementary Figure 6

Supplementary Figure 7

Supplementary Figure 8

Peer Review

Source Data

## Data Availability

The authors declare that the data supporting this study are available within the paper and its Supplementary Information. All other data are available from the authors upon reasonable request. The source data underlying Figs. 1 to 7, and Supplementary Figures 1 to 7 are provided as a Source Data file.
