# Supplementary material for: The Tec kinase ITK is essential for ILC2 survival and epithelial integrity in the intestine
Source: Nat Commun. 2019 Feb 15;10:784. doi: 10.1038/s41467-019-08699-9 (PMC6377622; doi:10.1038/s41467-019-08699-9)
Supplement: Supplementary file 1 — Supplementary Information [file 41467_2019_8699_MOESM1_ESM.pdf]

Supplementary Information

The Tec kinase ITK is essential for ILC2 survival and epithelial integrity in the intestine

Cho *et al.*

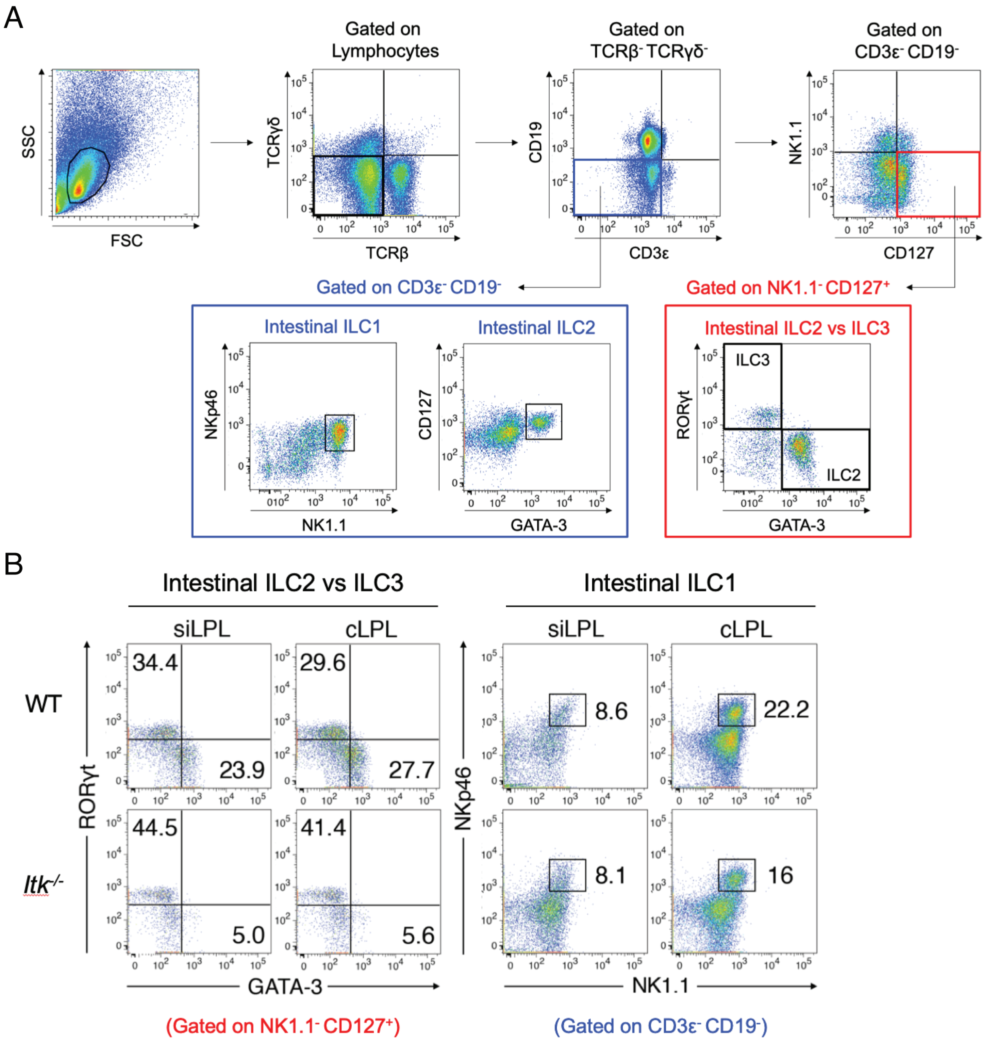

Supplementary Figure 1. Gating strategy used to identify multiple ILC subsets in the intestine

(A) Isolated lymphocytes from the BM, mLN, lung, and small and large intestine were stained with antibodies to identify multiple ILCs. From the dump gates (blue box), ILC1 and ILC2 were identified. Cells from the dump gates were further gated on NK1.1^{-} CD127^{+} cells (red box), and ILC2 and ILC3 are identified. (B) Based on this gating strategy, ILC2 vs ILC3 (left) and ILC1 from WT and *Itk*$^{-/-}$ mice are shown. Source data are provided as a Source Data file.

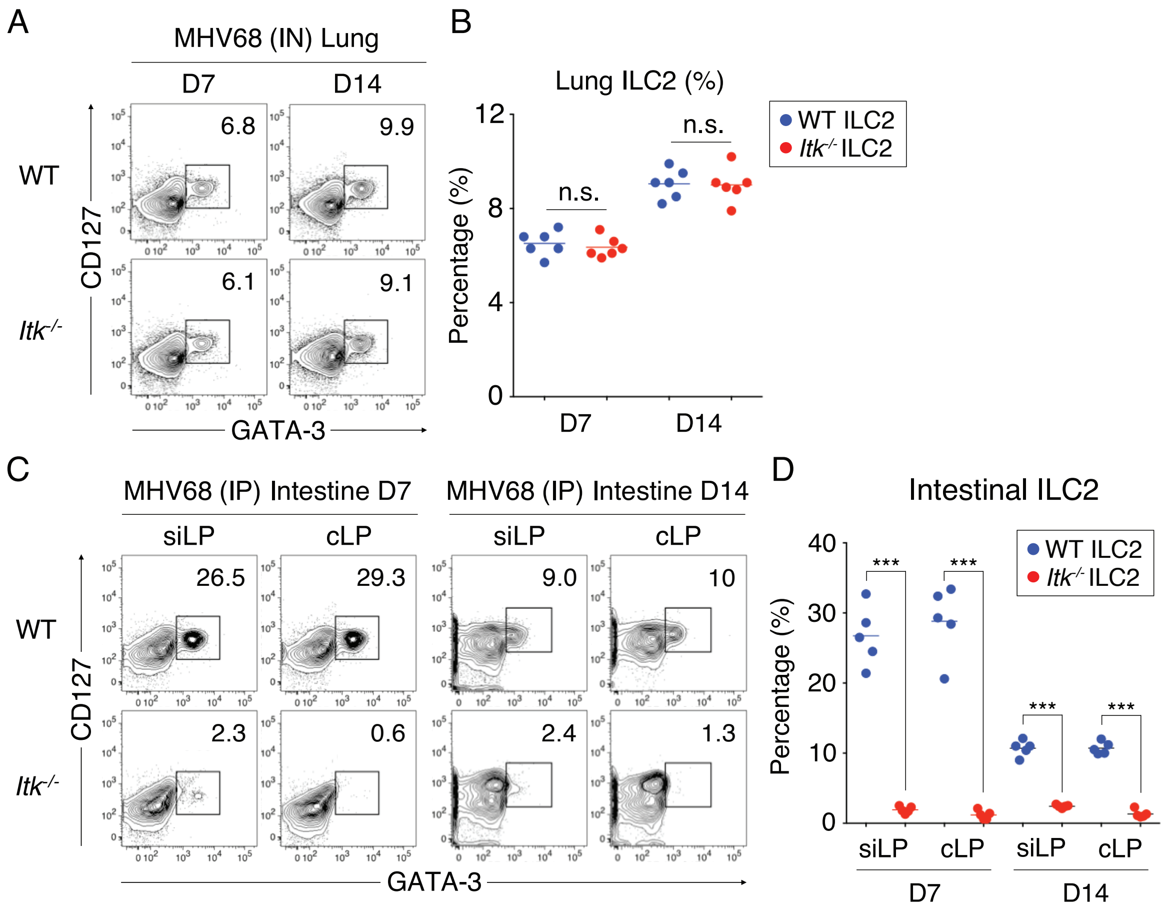

Supplementary Figure 2. Impaired expansion of intestinal ILC2 in virus-infected *Itk*$^{-/-}$ mice

(A-B) Lung ILC2 in WT and *Itk*$^{-/-}$ mice at D7 and D14 of post-infection with MHV68 (IN, 10^3 PFU/mouse). (C-D) Intestinal ILC2 from siLP or cLP of WT and *Itk*$^{-/-}$ mice at D7 and D14 with MHV68 (IP, 10^6 PFU/mouse). Data are the compilation of 2 independent experiments using WT (n=5-6) and *Itk*$^{-/-}$ mice (n=5-6). Statistical significance was analyzed using Student's *t* tests (***, $p < 0.001$). Source data are provided as a Source Data file.

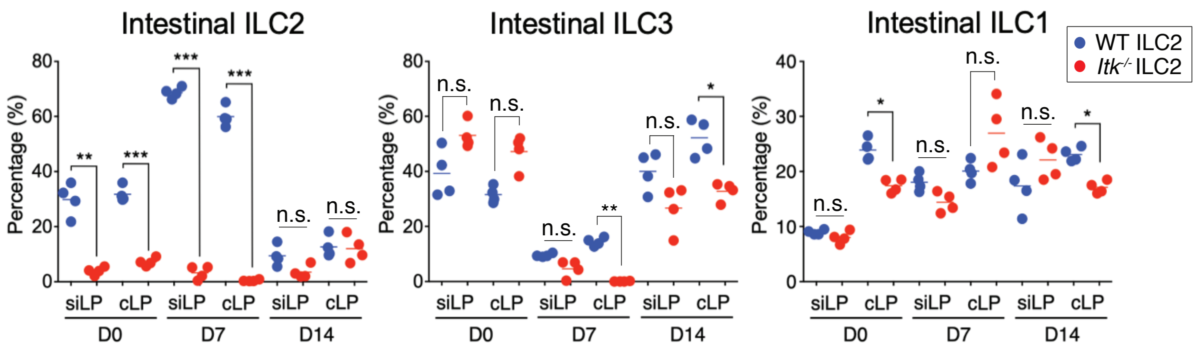

Supplementary Figure 3. *Itk$^{-/-}$* gut ILC2 are impaired in response to MHV68

(A-C) Intestinal ILC subsets were analyzed before (D0) and after MHV68 intraperitoneal infection (D7 or D14). Gating strategies are as shown in Figure 1F. Data are the compilation of 2 independent experiments using WT (n=4) and *Itk$^{-/-}$* mice (n=4). Statistical significance was analyzed using Student's *t* tests (*, $p < 0.05$; **, $p < 0.01$; ***, $p < 0.001$). Source data are provided as a Source Data file.

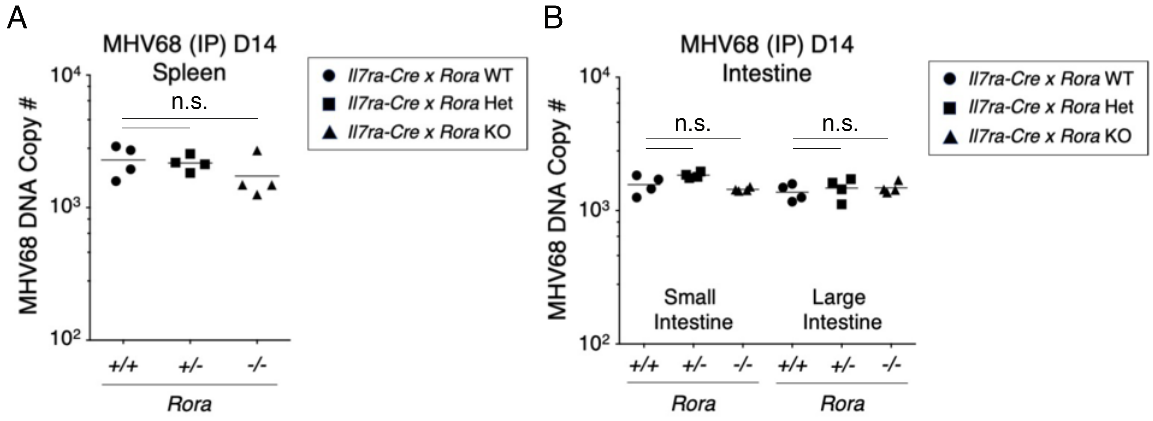

Supplementary Figure 4. ILC2 deficiency does not affect MHV68 control in the gut

(A-B) Viral copy numbers of MHV68 DNA in the spleen (A) and the small or large intestine (B) of littermates that were *Rora*$^{+/+}$ (n=4), *Rora*$^{+/fl}$ (n=4), and *Rora*$^{fl/fl}$ (n=4) crossed to *Il7ra-Cre* are shown at D14 post-infection. Data are the compilation of 2 independent experiments. Statistical significance was analyzed using Student's *t* tests. Source data are provided as a Source Data file.

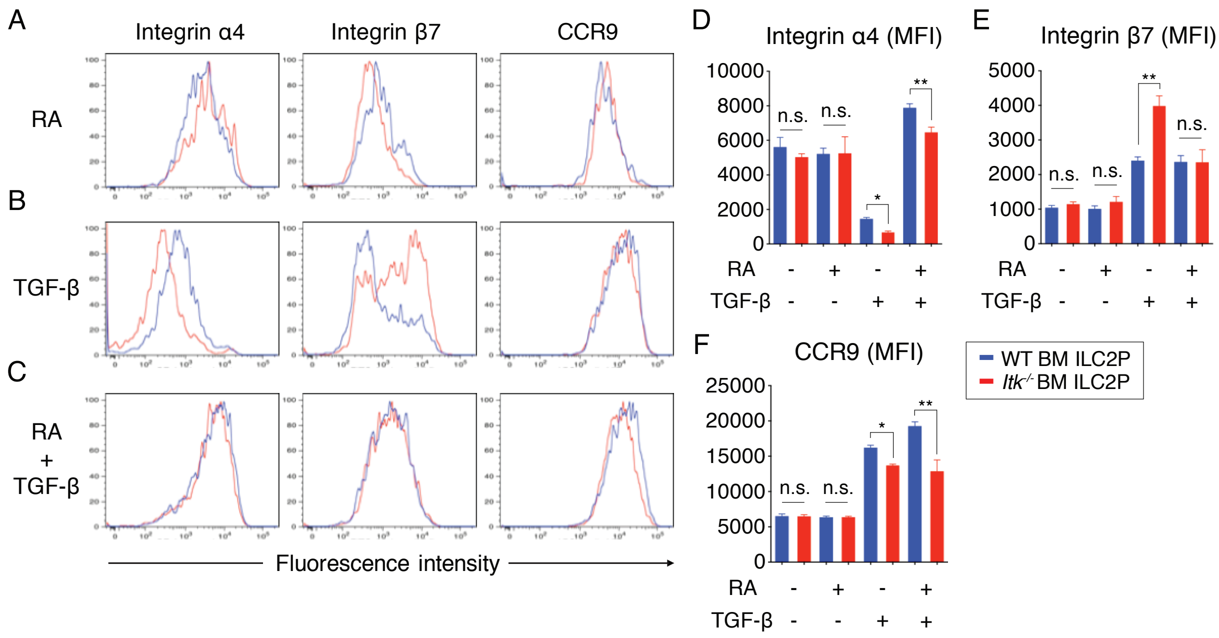

Supplementary Figure 5. ITK is not required for gut-homing receptor expression on BM ILC2P

(A-F) Lin$^-$ BM cells were cultured for 5 d *in vitro* with IL-2, IL-7, and IL-33 in the presence of RA and/or TGF-β. Levels of integrin α4, integrin β7, and CCR9 on cultured BM ILC2P with RA alone, TGF-β, or RA plus TGF-β are shown (A-C). Data are the compilation of 2 independent experiments using WT (n=4) and *Itk*$^{-/-}$ mice (n=4). Bar graphs with error bars show the averages and the SEMs. Statistical significance was analyzed using Student's *t* tests (*, $p < 0.05$; **, $p < 0.01$; ***). Source data are provided as a Source Data file.

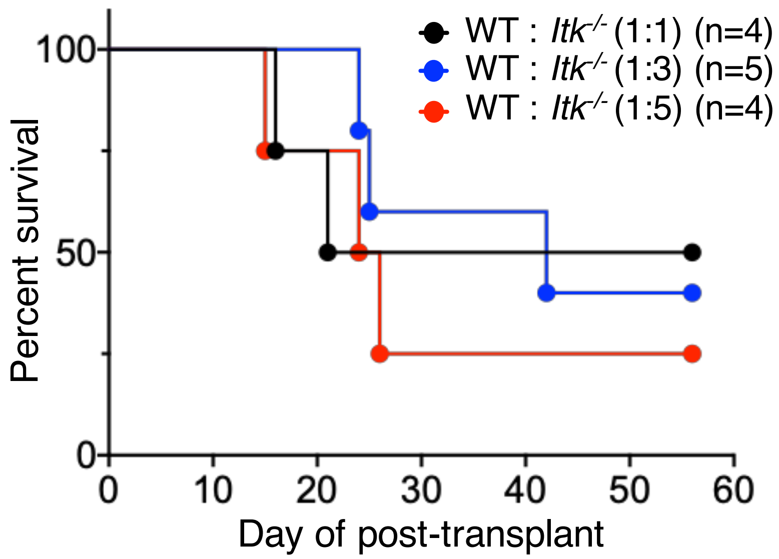

Supplementary Figure 6. WT and *Itk*$^{-/-}$ BM chimera recipients develop a lethality

Percent survival of the recipients of BM chimera with different WT and *Itk*$^{-/-}$ ratios (1:1, 1:3, and 1:5) are shown. Shown data are from the compilation of 2 independent experiments using 4-5 recipients per group. Source data are provided as a Source Data file.

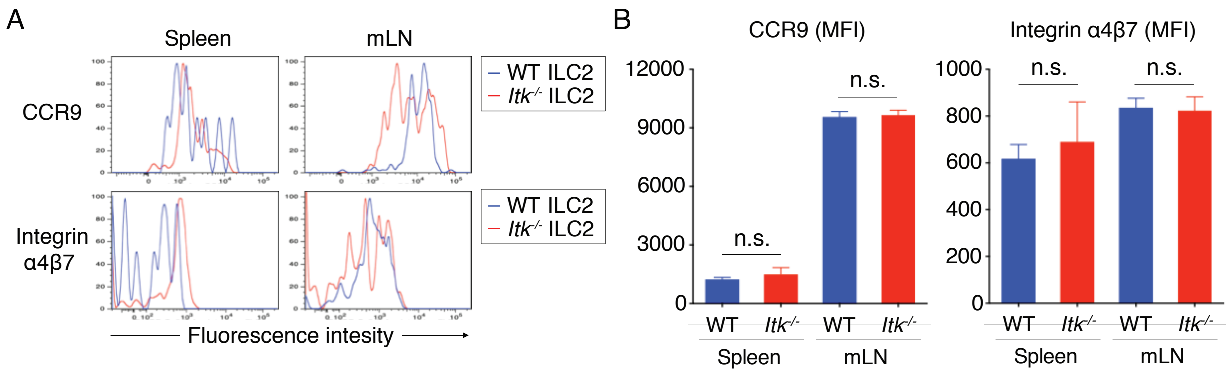

Supplementary Figure 7. ITK does not affect gut-homing receptors on transferred ILC2

(A-B) The expression of CCR9 and integrin α4β7 on adoptively transferred ILC2 in the spleen and mLN of *Rag1$^{-/-}$ Il2rg$^{-/-}$* hosts at D3 of post-transfer (A). Compilation of mean fluorescence of CCR9 and integrin α4β7 from each group of mice (B). Data are the compilation of 2 independent experiments using WT (n=4) and *Itk$^{-/-}$* mice (n=4). Bar graphs with error bars show the averages and the SEMs. Statistical significance was analyzed using Student's *t* tests. Source data are provided as a Source Data file.

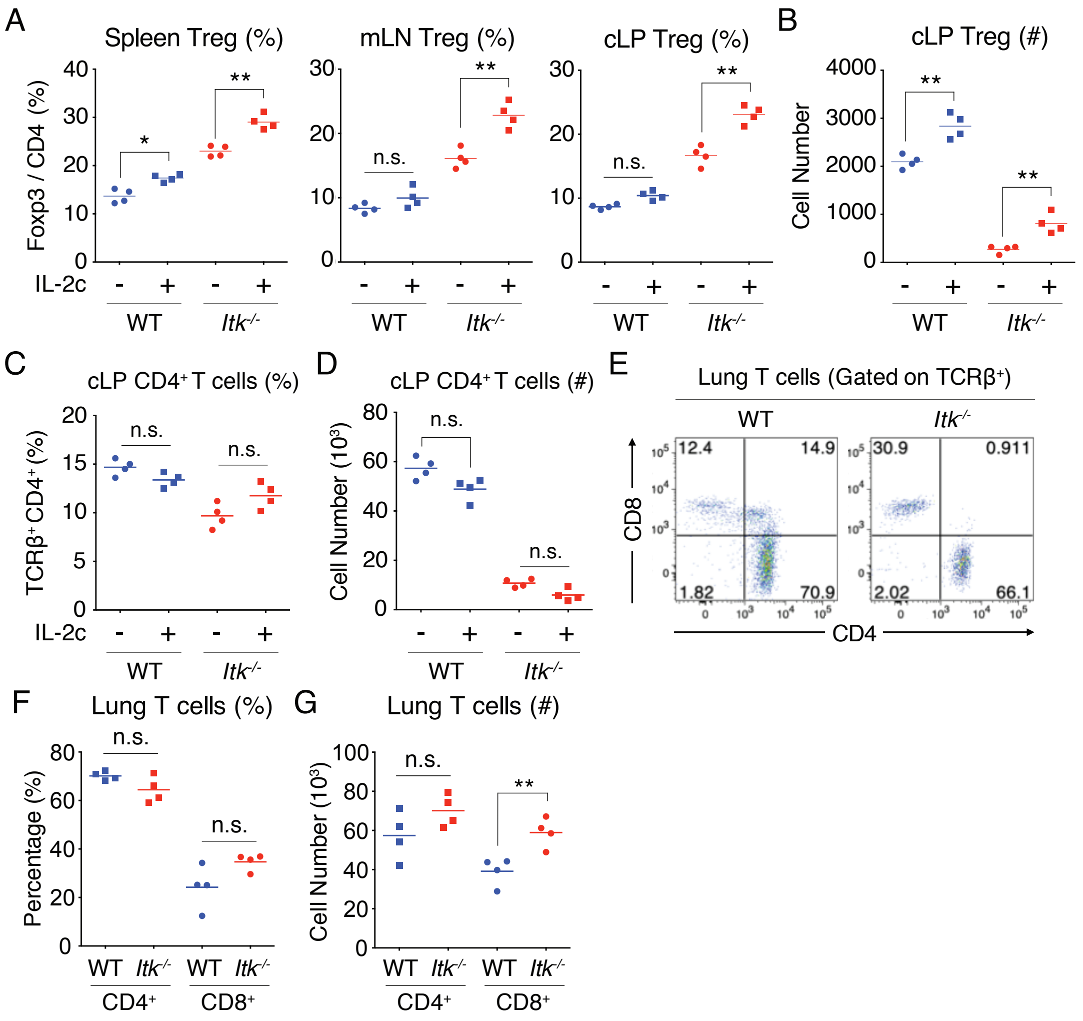

Supplementary Figure 8. IL-2c effect on CD4+ T cells and lung T cells in *Itk-/-* mice

(A-B) Treg (CD4+ Foxp3+) frequency from the spleen, mLN, and cLP from the PBS- and IL-2c-treated WT or *Itk-/-* mice is shown (A). cLP Treg number is enumerated (B). (C-D) The frequency (C) and the number (D) of cLP CD4+ Foxp3- T cells from PBS- and IL-2c-treated WT or *Itk-/-* mice are shown. (E-G) Lung T cell proportion (E and F) and the number (G) in naïve WT and *Itk-/-* mice are shown. Data are the compilation of 2-3 independent experiments using WT (n=4) and *Itk-/-* mice (n=4). Statistical significance was analyzed using Student's *t* tests (*, $p < 0.05$; **, $p < 0.01$; ***). Source data are provided as a Source Data file.
