# Supplementary figures and images for: The Tec kinase ITK is essential for ILC2 survival and epithelial integrity in the intestine
Source: Nat Commun. 2019 Feb 15;10:784. doi: 10.1038/s41467-019-08699-9 (PMC6377622; doi:10.1038/s41467-019-08699-9)

# Supplementary Figure 1

A

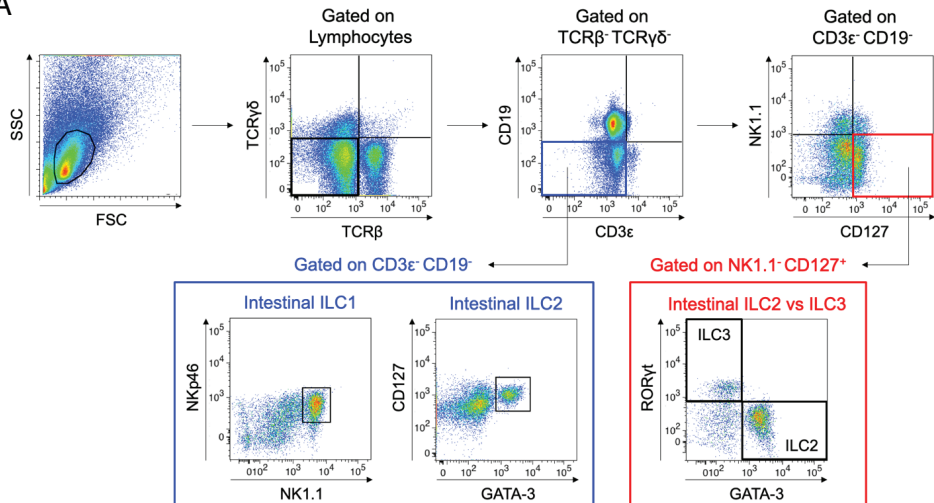

B

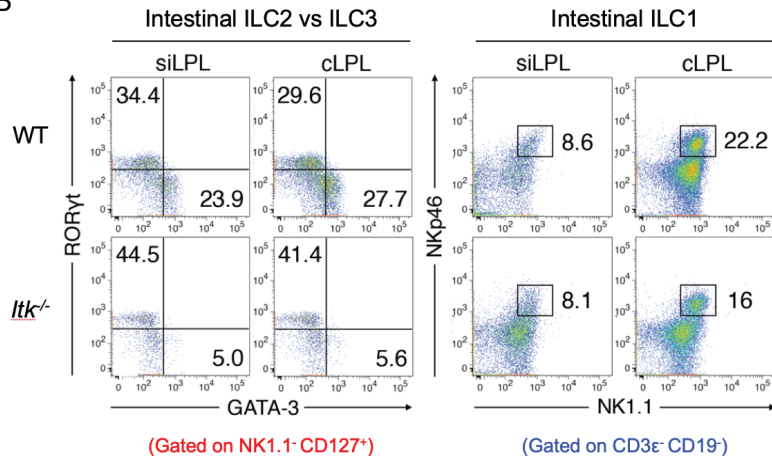

Supplement: Supplementary file 2 — Supplementary Figure 1 [file 41467_2019_8699_MOESM2_ESM.pdf]

# Supplementary Figure 2

A

MHV68 (IN) Lung

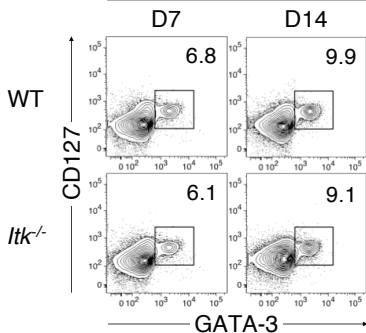

B

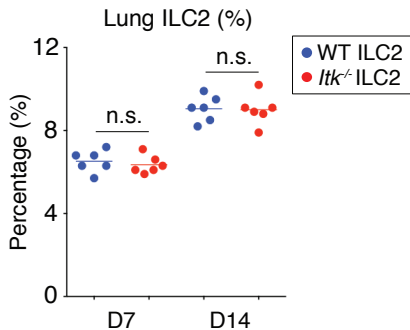

C

MHV68 (IP) Intestine D7

MHV68 (IP) Intestine D14

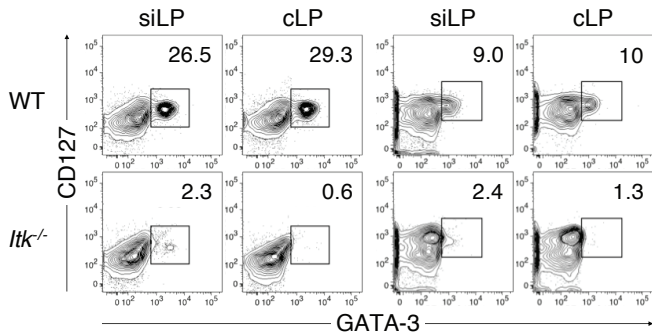

D

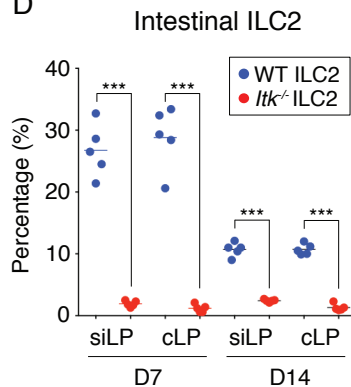

Supplement: Supplementary file 3 — Supplementary Figure 2 [file 41467_2019_8699_MOESM3_ESM.pdf]

# Supplementary Figure 3

## Intestinal ILC2

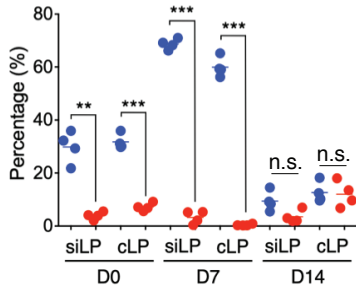

## Intestinal ILC3

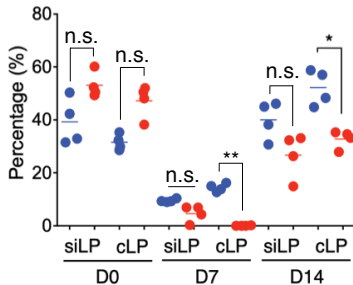

## Intestinal ILC1

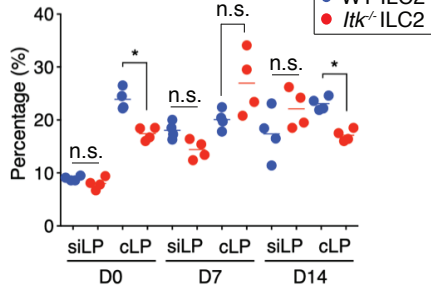

Supplement: Supplementary file 4 — Supplementary Figure 3 [file 41467_2019_8699_MOESM4_ESM.pdf]

# Supplementary Figure 4

A

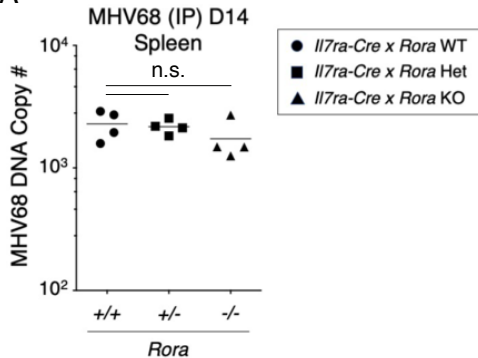

B

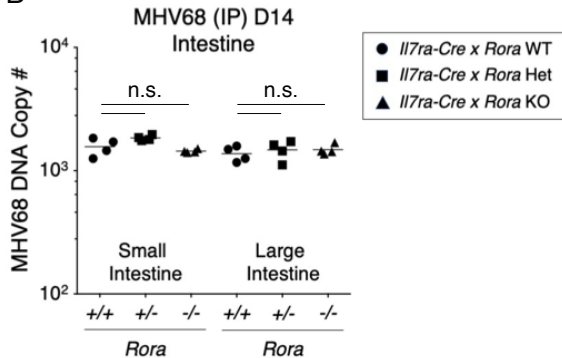

Supplement: Supplementary file 5 — Supplementary Figure 4 [file 41467_2019_8699_MOESM5_ESM.pdf]

# Supplementary Figure 5

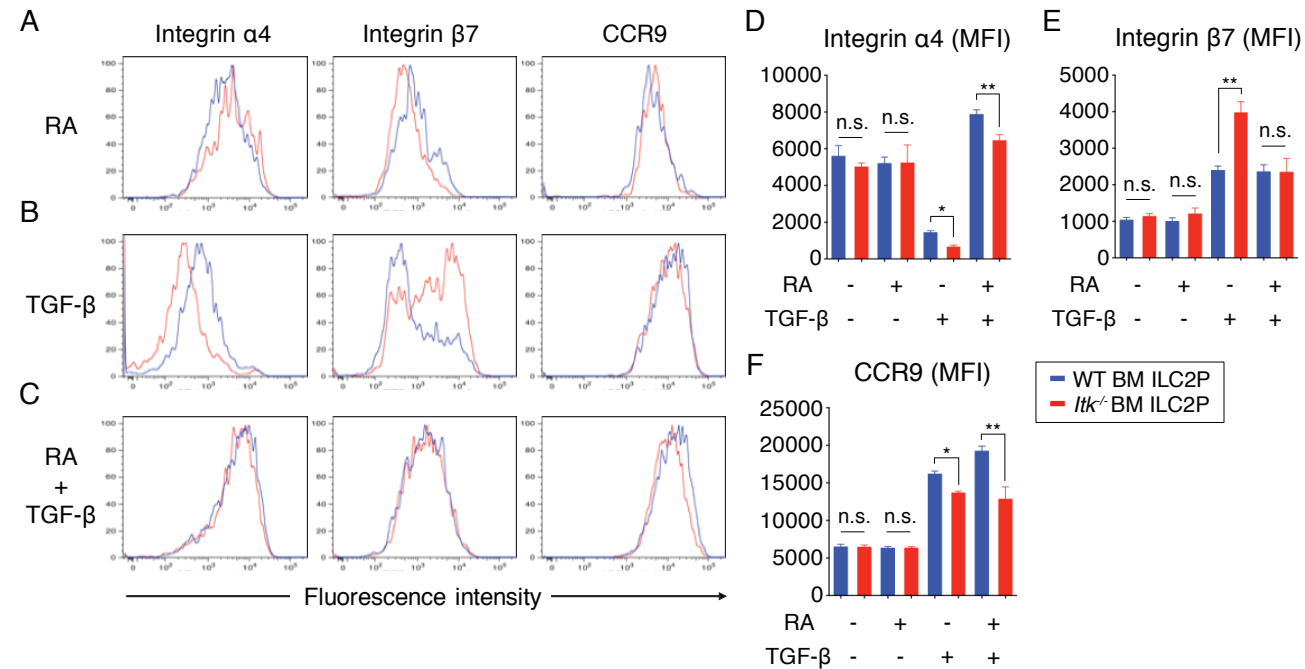

Supplement: Supplementary file 6 — Supplementary Figure 5 [file 41467_2019_8699_MOESM6_ESM.pdf]

## Supplementary Figure 6

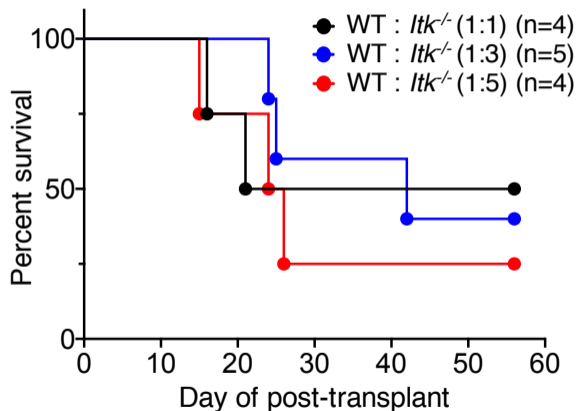

Supplement: Supplementary file 7 — Supplementary Figure 6 [file 41467_2019_8699_MOESM7_ESM.pdf]

# Supplementary Figure 7

A

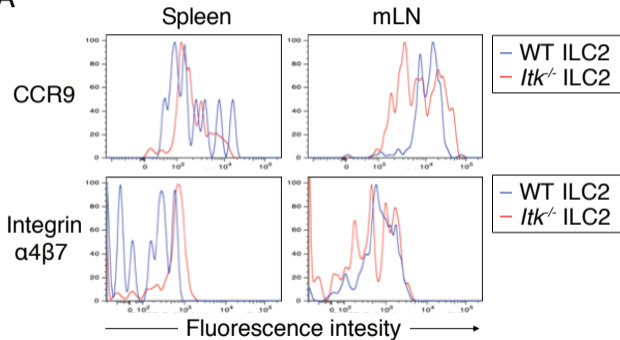

B

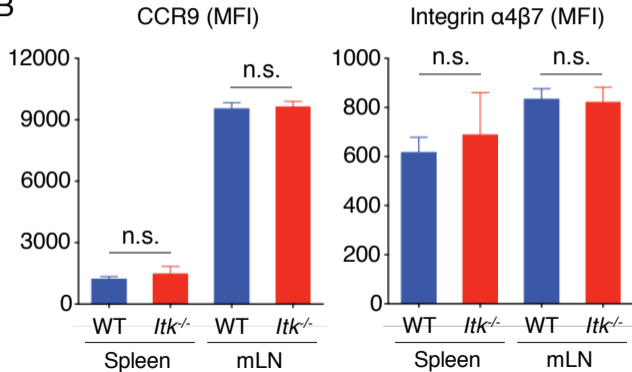

Supplement: Supplementary file 8 — Supplementary Figure 7 [file 41467_2019_8699_MOESM8_ESM.pdf]

# Supplementary Figure 8

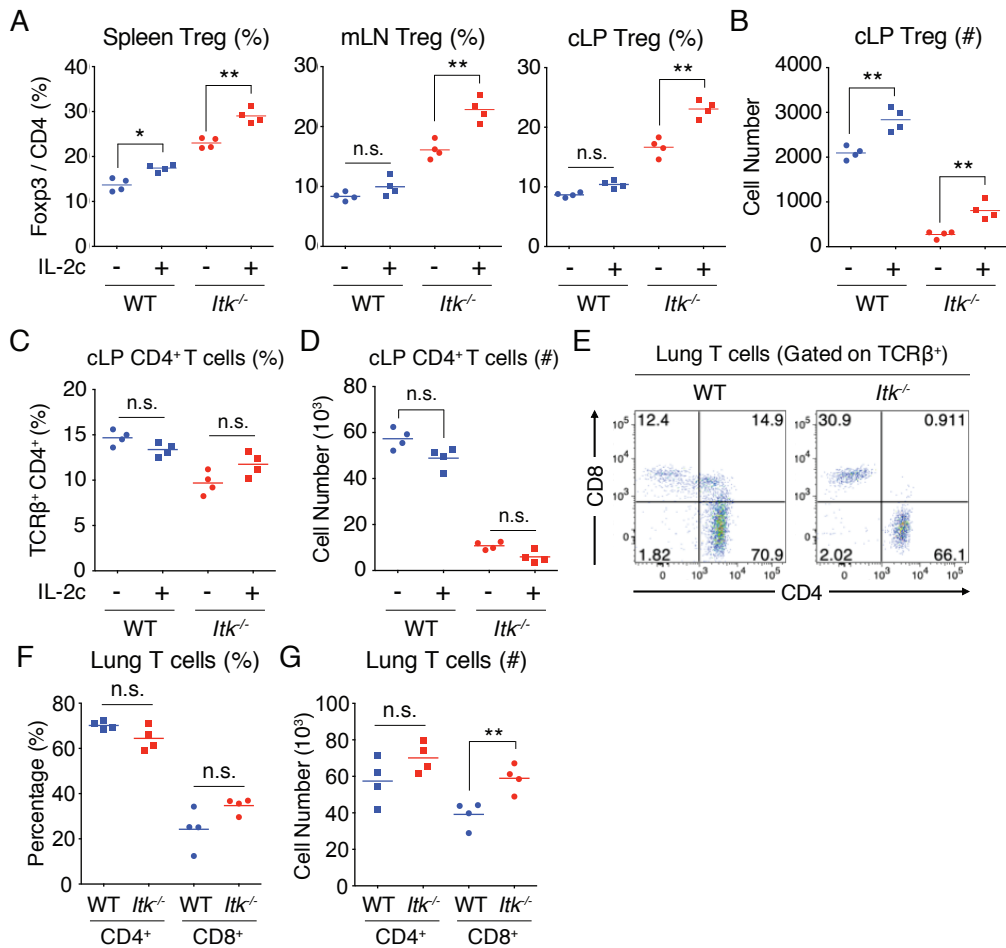

Supplement: Supplementary file 9 — Supplementary Figure 8 [file 41467_2019_8699_MOESM9_ESM.pdf]
