# Supplementary material for: The Tec kinase ITK is essential for ILC2 survival and epithelial integrity in the intestine
Source: Nat Commun. 2019 Feb 15;10:784. doi: 10.1038/s41467-019-08699-9 (PMC6377622; doi:10.1038/s41467-019-08699-9)
Supplement: Supplementary file 10 — Peer Review [file 41467_2019_8699_MOESM10_ESM.pdf]

Reviewers' comments:

Reviewer #1, expert in lymphocyte signaling (Remarks to the Author):

The Tec tyrosine kinase ITK is an important signaling molecule in both adaptive and, as has been more recently shown, innate immunity. The role of ITK in the function and development of cells of adaptive immunity (particularly CD4-T cells) has been extensively studied. Recently, ITK has been shown to be also expressed in Innate Lymphoid Cells (ILC) with significantly higher levels in ILC2. However, the role of this tyrosine kinase in ILC2 is not clearly understood. This has prompted the present investigation by Hyoung-Soo et al.

The manuscript starts with a very good and comprehensive introduction of the topic leading to a clear statement of the questions to be addressed. The experimental approach is sound, clearly described, and the data with the drawn conclusions are novel and provide strong evidence for the authors' interpretations. A strength in the presentation of the data is the inclusion of logical and concise summaries and interpretations at the end of each section of the 'Results'. Even though several of the experiments presented include negative data (no differences between WT ITK -/- groups), they nevertheless provide for a logical progression of the authors' thinking process.

Previous reports have demonstrated the presence of ITK in ILC's particularly ILC2, but the functional significance of ITK in these cells is not understood. Even though the present work has not uncovered the actual signaling mechanism of ITK's function in ILC2, its role in intestinal ILC2 survival is a novel finding. The role of tyrosine kinases in intestinal integrity, particularly in intestinal ILC2, has not been previously investigated and thus, the present report extend this particular field significantly. Furthermore, the findings should be of interest to investigators in the broader field of tyrosine kinases and innate immunity.

Specific comments on figures:

Fig 4 A-B presents evidence that ITK-/- ILC2 express normal gut-homing receptors. This is informative and relevant. However, in view of previously published report (ref 24 in manuscript), the rest of data in this Figure (panels C-H) displaying the role of ITK in regulating gut-homing receptors in response to RA and TGFß are rather predictable. For the sake of brevity, I would suggest eliminating panels C-H and simply mentioning the results as data not shown or, alternatively, include these data in the supplementary section.

In Fig 6 the data show that even though CCR9 expression was significantly reduced in sILP of Itk -/- ILC2, the same was not observed in cLP. This phenomenon was not seen with Integrin α4β7. Any hypothetical explanation for this result?

In Fig 7 data display interesting differences in IL-13 and IL-5 production between WT and Itk -/- mice. Have the authors looked for this phenomenon in sILP? or is it technically difficult to do this?

A clarification would be helpful in the inset of panel D, Fig 8. It is not clear what the lower box with the asterisks refers to. In addition, there is a panel mislabeling in this figure; there are two panels E but not panel H as it is referred to in the text. Letters shifted?

Reviewer #2, expert in intestinal ILC biology (Remarks to the Author):

Cho et al. examined the role of ITK in ILC2 homeostasis. The authors confirm previous results (Gomez-Rodriguez et al., 2016) showing that steady state ILC2 homeostasis in the lung is not altered in Itk-/- mice. Furthermore, Cho et al. demonstrate that that Itk-/- and WT mice have comparable numbers of ILC2 in their BM and mLN. However, frequencies and numbers of ILC2 are selectively

reduced in the intestine of Itk-/- mice while ILC1 and ILC3 remained largely unaffected. Upon viral infection, the authors observed ILC2 expansion in the lung, but not the intestine, of Itk-/- mice. Furthermore, the authors excluded a major impact of ITK on the expression of known gut homing receptors by BM ILC2p/intestinal ILC2s. Additionally, in vitro experiments with lin- BM cells proved IL-33 responsiveness of Itk-/- cells. On the contrary, IL-33 administration failed to expand ILC2 in the intestine, but not mLN and PEC, of Itk-/- mice again arguing for a selective effect of ITK in intestinal ILC2. Despite mild effects of ITK on known intestinal homing receptors, adoptive transfers of WT and Itk-/- ILC2p into Rag1-/-IL-2rg-/- revealed that Itk-/- ILC2 failed to accumulate in the intestine. Furthermore, Itk-/- mice proved to be more sensitive to DSS-induced tissue damage, a response that could be ameliorated by IL-2c administration, which restored ILC2 numbers in the intestine of Itk-/- mice. Frequencies of Annexin V+ ILC2 were elevated in the small intestine, but not the colon, of IL-2c-treated Itk-/- mice. In the steady state, however, Annexin V levels of intestinal ILC2 did not differ between WT and Itk-/- mice, despite significantly reduced levels of anti-apoptotic Bcl-2 in the latter. Based on these findings the authors conclude that "that the defect in intestinal ILC2 in Itk-/- mice results from impaired ILC2 survival in the intestinal environment" (p. 17).
Although it is interesting that Itk-/- mice show an intestine-specific reduction of ILC2, the molecular basis of this phenomenon remains largely unclear. Furthermore, it is questionable whether the experimental systems are suited to draw the conclusion summarized in the title of this manuscript.

The following specific concerns are raised:
1. In Fig. 1 the authors show a selective ILC2 defect in the intestine, but not the lung, of Itk-/- mice. Based on these findings it is difficult to understand why the authors decided to present data on ILC2 responses in virus-infected lungs next (Fig. 2). These data should be moved to the supplement.
2. Viral infection of Rorafl/fl x IL7ra-Cre mice showed that ILC2-deficiency does not affect viral clearance in the intestine (Fig. S3). In light of these findings it is difficult to comprehend why intestinal ILC2 responses of virally infected Itk-/- mice are shown in Fig. 2. If the only purpose is to demonstrate "that Itk-/- mice have an intestinal ILC2 deficit that cannot be overcome by a lytic viral insult to the GI epithelium" (p. 9) it would be sufficient to show these results in the supplement.
3. On page 13 the authors state "Itk-deficient ILC2 are impaired in migration to the intestine". In the discussion (p. 19), however, they state: "the intestinal ILC2 defect in Itk-/- mice is unlikely to be accounted for by a cell-intrinsic migration defect, despite the reduction in Itk-/- ILC2P seen three days after transfer of ILC2 precursors into Rag1-/-Il2rg-/- hosts." The authors should resolve this contradiction.
4. On page 14 of their manuscript the authors ask "whether the intestinal ILC2 defect in Itk-/- mice affected gastrointestinal tissue integrity and repair from intestinal damage". In their related manuscript, however, the authors demonstrate that gut-resident T cell numbers are strongly reduced in Itk-/- mice. This finding adds to the many reports demonstrating fundamental effects of ITK on T cell development and function. Although this reviewer agrees that T cells are dispensable for the induction of DSS colitis, it cannot be excluded that altered T cell homeostasis/function in Itk-/- mice promotes disease severity independent of ILC2-deficiency (Fig. 7). To clarify whether there is a role of ITK-dependent ILC2 in the control of DSS colitis at all, the authors should study DSS colitis in ILC2-deficient Rorafl/fl x IL7ra-Cre mice, given their T cell are indeed normal.
5. Adding to the point above, it cannot be excluded that the steady state defect of intestinal ILC2 homeostasis in Itk-/- mice results from ILC2-idependent ITK effects. To exclude such environmental effects, the authors should study ILC2 homeostasis in e.g. mixed bone marrow chimeras. To ensure normal immune cell development they could mix BM of Rorafl/fl x IL7ra-Cre mice with BM of e.g. Rag-/-Itk-/- mice. Alternatively, ILC2-specific Itk-knockout mice could be used. Transfers of ILC2p into Rag1-/-Il2rg-/- mice are difficult to interpret since the cytokine environment is severely altered in these mice (Ramsey et al., 2008).
6. Labeling of Fig. 8 is not correct (E is shown twice). Furthermore, in Fig. 8E and 8F all histograms are labeled with "siLP". According to the legend cLP ILC2 should be displayed as well.
7. IL-2c treatment alters T cell homeostasis in mice (Boyman et al., 2006). Does the expansion of intestinal ILC2 in IL-2c-treated Itk-/- mice result from a normalization of T cell homeostasis (allowing the restoration of IL-2-independent T-ILC2 interactions) or does it result form the direct action of IL-

2c on ILC2? This question should be addressed experimentally, especially because the authors "speculated that the availability of IL-2 from T cells in the gut microenvironment of Itk-/- mice would likely be limited leading to disruption of ILC2 tissue homeostasis." To support this point, experiments analyzing intestinal Itk-/- ILC2 in a normal T cell-competent environment (see mixed BM chimeras above) are required.

8. The authors measured Annexin V levels to visualize cell death after IL-2c treatment (Fig. 8E). How do the authors explain that death rates of intestinal ILC2 do not differ between untreated WT and Itk-/- mice although ILC2 numbers are strongly reduced in the latter?

References

Boyman, O., Kovar, M., Rubinstein, M.P., Surh, C.D., and Sprent, J. (2006). Selective stimulation of T cell subsets with antibody-cytokine immune complexes. Science 311, 1924–1927.

Gomez-Rodriguez, J., Meylan, F., Handon, R., Hayes, E.T., Anderson, S.M., Kirby, M.R., Siegel, R.M., and Schwartzberg, P.L. (2016). Itk is required for Th9 differentiation via TCR-mediated induction of IL-2 and IRF4. Nature Communications 7, 10857.

Ramsey, C., Rubinstein, M.P., Kim, D.M., Cho, J.-H., Sprent, J., and Surh, C.D. (2008). The lymphopenic environment of CD132 (common gamma-chain)-deficient hosts elicits rapid homeostatic proliferation of naive T cells via IL-15. J Immunol 180, 5320–5326.

Reviewer #3, expert in intestinal lymphocytes (Remarks to the Author):

In "The Tec kinase ITK promotes intestinal tissue integrity via regulation of ILC2," Cho and her colleagues examine the role of the Tec kinase, Itk, in the homeostasis of ILC2's in the gut. They report that whole animal deletion of Itk is associated with significant decrease in numbers of ILC2's, but only in the small intestinal lamina propria. They find no evidence for decreased expression of intestine-specific chemokine or integrin receptors, or failure to respond to IL-33. They note that this is associated with disrupted intestinal integrity with increased permeability and increased morbidity and mortality following DSS colitis. There is a significant effort made to determine if there is a developmental defect that inhibits migration into the BM; however, the BM compartment looks normal. Instead, the authors suggest the ILC2 defect is secondary to a decrease in the number of conventional T cells and a lack of IL-2. Thus, they show that treatment of itk-deficient mice with IL-2-anti-IL-2 complexes rescues the compartment.

The manuscript is easy to read and the figures are quite clear.

This is a very interesting observation. However, my primary concern is that it doesn't explain the tissue specificity of the ILC2 deficit.

Do the ILC2's in other tissues express itk?

Are there normal numbers of conventional T cells in other tissues? If yes, this would explain the intestine specificity. If not, then it is not the explanation.

Does infection not increase the number of local T cells? Or is the increase not adequate to generate IL-2?

A second question: the authors suggest that the phenotype is cell intrinsic, given the similar finding in transfers to RAG-deficient mice. If however, the effect is due to inadequate T cell IL-2, can it be corrected with normal T cells? I can think of two ways to ask this question.

If itk-/- BM progenitors are transferred into mice with WT T cells, do normal numbers end up in the small intestine?

A cleaner experiment: in a mixed BM chimera, are the intestinal ILC2's equally WT and itk-deficient?

Additional questions:

Given the phenotype of DSS, as well as, the intestinal permeability defect, do itk-deficient ILC2s express normal levels of amphiregulin? Is that concretely what's lacking in the intestine?

The authors point out that the ILC2s that make it to the intestine have normal levels of IL-33R and other receptors. However, this would be expected for those cells that do home there—the difficulty is in determining the phenotype of those that don't.

Where does the death occur in the absence of IL-2? Do cells not make it to the intestine? Do they enter with normal chemokine receptor and integrin expression and then die? (The latter is what is implied by the interpretation proferred.)

Point-By-Point Response to Reviewers

We thank the reviewers for their thoughtful and helpful comments. Below, please find a point-by-point response to these questions and concerns. All reviewer comments are in black font. Our responses to these concerns are all in blue font.

Reviewer #1 Comments

The Tec tyrosine kinase ITK is an important signaling molecule in both adaptive and, as has been more recently shown, innate immunity. The role of ITK in the function and development of cells of adaptive immunity (particularly $CD4^+$ T cells) has been extensively studied. Recently, ITK has been shown to be also expressed in innate lymphoid cells (ILC) with significantly higher levels in ILC2. However, the role of this tyrosine kinase in ILC2 is not clearly understood. This has prompted the present investigation by Cho *et al.*

The manuscript starts with a very good and comprehensive introduction of the topic leading to a clear statement of the questions to be addressed. The experimental approach is sound, clearly described, and the data with the drawn conclusions are novel and provide strong evidence for the authors' interpretations. A strength in the presentation of the data is the inclusion of logical and concise summaries and interpretations at the end of each section of the 'Results'. Even though several of the experiments presented include negative data (no differences between WT and *Itk*$^{-/-}$ groups), they nevertheless provide for a logical progression of the authors' thinking process.

Previous reports have demonstrated the presence of ITK in ILC's particularly ILC2, but the functional significance of ITK in these cells is not understood. Even though the present work has not uncovered the actual signaling mechanism of ITK's function in ILC2, its role in intestinal ILC2 survival is a novel finding. The role of tyrosine kinases in intestinal integrity, particularly in intestinal ILC2, has not been previously investigated and thus, the present report extends this particular field significantly. Furthermore, the

findings should be of interest to investigators in the broader field of tyrosine kinases and innate immunity.

Specific Comments of Figures:

1. Fig. 4A-B presents evidence that *Itk*$^{-/-}$ ILC2 express normal gut-homing receptors. This is informative and relevant. However, in view of previously published report (Reference 24 in the manuscript), the rest of data in this figure (panels C-H) displaying the role of ITK in regulating gut-homing receptors in response to RA and TGF-β are rather predictable. For the sake of brevity, I would suggest eliminating panels C-H and simply mentioning the results as data not shown or, alternatively, include these data in the supplementary section.

As suggested above, Fig. 4C-H are now moved to the supplementary data (Fig. S5A to F).

2. In Fig. 6, the data show that even though CCR9 expression was significantly reduced in sILP of *Itk*$^{-/-}$ ILC2, the same was not observed in cLP. This phenomenon was not seen with Integrin α4β7. Any hypothetical explanation for this result?

It is not clear why the reduced CCR9 expression was only observed on siLP Itk$^{-/-}$ ILC2, but not on cLP Itk$^{-/-}$ ILC2. Based on a comparable level of CCR9 expression in both WT and Itk$^{-/-}$ ILC2 when they are stimulated in vitro with RA and TGF-β, we assume this change in their expression is happening after the cells have migrated into the gut tissue. As shown in our related manuscript, the deficit in T cells is more pronounced in the small intestine compared with the colon. Therefore, it is possible that IL-2 availability will be more limited in the small intestine, and this might be related to CCR9 expression and the survival of ILC2.

Regarding integrin α4β7 expression, the requirement for this receptor is at the initial stage of cell entry into the gut mucosa, rather than for retention. The timepoint used in this experiment was D3 of post-transfer. It is possible that at this time the cells are downregulating integrin α4β7 expression after entering the lamina propria. This is consistent with our finding that analysis of transferred cells recovered from the spleen and mLN did not show any reduced α4β7 expression on Itk$^{-/-}$ versus WT ILC2. These data are now included in the revised manuscript as Fig. S6. Although we have not

examined other time points to assess expression levels in the intestinal tissue at earlier times, but we did observe that the expression of α4β7 on these cells analyzed at D3 post-transfer is much lower than the levels observed prior to the transfer.

3. In Fig. 7, the data display interesting differences in IL-13 and IL-5 production between WT and *Itk*$^{-/-}$ mice. Have the authors looked for this phenomenon in siLP? or is it technically difficult to do this?

The major reason we did not look into ICS data of siLP ILC in Fig. 7 was due to the effect of DSS on the colon, not on the small intestine. Although we did not check the cytokine production of siLP ILC in DSS-induced colitis, we observed that Itk$^{-/-}$ *ILC2 isolated under steady-state conditions showed impaired IL-5 and IL-13 production in response to ex vivo stimulation with PMA and Ionomycin. These data are now included in the revised manuscript in Figure 1C and D.*

4. A clarification would be helpful in the inset of panel D, Fig 8. It is not clear what the lower box with the asterisks refers to. In addition, there is a panel mislabeling in this figure; there are two panels E but not panel H as it is referred to in the text. Letters shifted?

We apologize for the error and lack of clarification of asterisks. The lower box shows the statistical analysis between the designated groups. To clarify this, we have added a second legend box to the figure panel indicating the comparison groups corresponding to the blue versus the red asterisks (Fig. 7D). In addition, the mislabeled figures are now revised in Fig. 7D to F.

Reviewer #2 Comments

Cho *et al.* examined the role of ITK in ILC2 homeostasis. The authors confirm previous results (Gomez-Rodriguez et al., 2016) showing that steady state ILC2 homeostasis in the lung is not altered in *Itk*$^{-/-}$ mice. Furthermore, Cho et al. demonstrate that that *Itk*$^{-/-}$ and WT mice have comparable numbers of ILC2 in their BM and mLN. However, frequencies and numbers of ILC2 are selectively reduced in the intestine of *Itk*$^{-/-}$ mice while ILC1 and ILC3 remained largely unaffected. Upon viral infection, the authors observed ILC2 expansion in the lung, but not the intestine, of *Itk*$^{-/-}$ mice. Furthermore, the authors excluded a major impact of ITK on the expression of known gut homing receptors by BM ILC2p/intestinal ILC2s. Additionally, in vitro experiments with lin- BM cells proved IL-33 responsiveness of *Itk*$^{-/-}$ cells. On the contrary, IL-33 administration failed to expand ILC2 in the intestine, but not mLN and PEC, of *Itk*$^{-/-}$ mice again arguing for a selective effect of ITK in intestinal ILC2.

Despite mild effects of ITK on known intestinal homing receptors, adoptive transfers of WT and *Itk*$^{-/-}$ ILC2p into *Rag1*$^{-/-}$ *Il2rg*$^{-/-}$ revealed that *Itk*$^{-/-}$ ILC2 failed to accumulate in the intestine. Furthermore, *Itk*$^{-/-}$ mice proved to be more sensitive to DSS-induced tissue damage, a response that could be ameliorated by IL-2c administration, which restored ILC2 numbers in the intestine of *Itk*$^{-/-}$ mice. Frequencies of Annexin V^{+} ILC2 were elevated in the small intestine, but not the colon, of IL-2c-treated *Itk*$^{-/-}$ mice. In the steady state, however, Annexin V levels of intestinal ILC2 did not differ between WT and *Itk*$^{-/-}$ mice, despite significantly reduced levels of anti-apoptotic Bcl-2 in the latter. Based on these findings the authors conclude that “that the defect in intestinal ILC2 in *Itk*$^{-/-}$ mice results from impaired ILC2 survival in the intestinal environment (p. 17)”.

Although it is interesting that *Itk*$^{-/-}$ mice show an intestine-specific reduction of ILC2, the molecular basis of this phenomenon remains largely unclear. Furthermore, it is questionable whether the experimental systems are suited to draw the conclusion summarized in the title of this manuscript.

We appreciate the reviewer’s comment. As the reviewer mentioned above, we agree the point that this manuscript lacks a molecular-based mechanism to explain ITK-driven defect ILC2. However, we tried to more focus on a cell-intrinsic defect of ITK on ILC2, which can be co-explained by cell survival and IL-2 tissue availability in the intestinal

tissue. In our revised manuscript, we confirmed that ILC2 homeostasis in the intestine was disrupted irrespective of T cells in Rag2$^{-/-}$ Itk$^{-/-}$ mice supporting the notion that ITK deficiency affects intestinal homeostasis of ILC2.

The following specific concerns are raised:

1. In Fig. 1, the authors show a selective ILC2 defect in the intestine, but not the lung, of *Itk*$^{-/-}$ mice. Based on these findings it is difficult to understand why the authors decided to present data on ILC2 responses in virus-infected lungs next (Fig. 2). These data should be moved to the supplement.

In the revised manuscript, Fig. 2 is now moved to the supplement (Fig. S2).

2. Viral infection of *Rora*$^{fl/fl}$ x *IL7ra-Cre* mice showed that ILC2 deficiency does not affect viral clearance in the intestine (Fig. S3). In light of these findings it is difficult to comprehend why intestinal ILC2 responses of virally infected *Itk*$^{-/-}$ mice are shown in Fig. 2. If the only purpose is to demonstrate “that *Itk*$^{-/-}$ mice have an intestinal ILC2 deficit that cannot be overcome by a lytic viral insult to the GI epithelium” (p. 9), it would be sufficient to show these results in the supplement.

As suggested, Fig. 2 has been moved to the supplement, and is now Fig. S2.

3. On page 13 the authors state “*Itk*-deficient ILC2 are impaired in migration to the intestine”. In the discussion (p. 19), however, they state: “the intestinal ILC2 defect in *Itk*$^{-/-}$ mice is unlikely to be accounted for by a cell-intrinsic migration defect, despite the reduction in *Itk*$^{-/-}$ ILC2P seen three days after transfer of ILC2 precursors into *Rag1*$^{-/-}$ *Il2rg*$^{-/-}$ hosts.” The authors should resolve this contradiction.

We apologize for the confusion. Our overall interpretation is that two components contribute together to account for the magnitude of the intestinal ILC2 deficit seen in the absence of ITK. One component is a cell-intrinsic function for ITK in the persistence of intestinal ILC2 and the second is a bystander role for IL-2, possibly produced by T cells. To clarify, the section of the text describing Fig. 5 is now revised with the title: “Itk has a cell intrinsic function in intestinal ILC2 homeostasis”.

4. On page 14 of their manuscript the authors ask "whether the intestinal ILC2 defect in *Itk*$^{-/-}$ mice affected gastrointestinal tissue integrity and repair from intestinal damage". In their related manuscript, however, the authors demonstrate that gut-resident T cell numbers are strongly reduced in *Itk*$^{-/-}$ mice. This finding adds to the many reports demonstrating fundamental effects of ITK on T cell development and function. Although this reviewer agrees that T cells are dispensable for the induction of DSS colitis, it cannot be excluded that altered T cell homeostasis/function in *Itk*$^{-/-}$ mice promotes disease severity independent of ILC2 deficiency (Fig. 7). To clarify whether there is a role of ITK-dependent ILC2 in the control of DSS colitis at all, the authors should study DSS colitis in ILC2-deficient *Rora*$^{fl/fl}$ x *IL7ra-Cre* mice, given their T cell are indeed normal.

We agree with the reviewer's comment that the DSS colitis experiment using ILC2-deficient mice would be useful to confirm the role of ILC2 on intestinal integrity, and this experiment would provide information to clarify the contribution of T cells versus ILC2 in the responses of Itk$^{-/-}$*mice to DSS. Unfortunately, we no longer have access to these mice to perform these studies. In light of this, we have included the caveat that we cannot rule out a contribution of T cells (or the absence of T cells) to the severity of the responses of Itk*$^{-/-}$*mice to DSS (p. 19).*

5. Adding to the point above, it cannot be excluded that the steady state defect of intestinal ILC2 homeostasis in *Itk*$^{-/-}$ mice results from ILC2-independent ITK effects. To exclude such environmental effects, the authors should study ILC2 homeostasis in e.g. mixed bone marrow chimeras. To ensure normal immune cell development they could mix BM of *Rora*$^{fl/fl}$ x *IL7ra-Cre* mice with BM of e.g. *Rag*$^{-/-}$ *Itk*$^{-/-}$ mice. Alternatively, ILC2-specific *Itk*-knockout mice could be used. Transfers of ILC2p into *Rag1*$^{-/-}$*Il2rg*$^{-/-}$ mice are difficult to interpret since the cytokine environment is severely altered in these mice (Ramsey *et al.*, 2008).

We thank the reviewer for these comments. As suggested, we have tried a mixed chimera experiment using WT and Itk$^{-/-}$ *BM mixed at several different ratios prior to reconstitution. Unfortunately, the majority of these mice did not survive the BM*

reconstitution, and the remaining number of mice was insufficient to allow us to make any significant conclusions.

As an alternative approach, we bred Rag2$^{-/-}$ mice with Itk$^{-/-}$ mice and examined Rag2$^{-/-}$ Itk$^{+/+}$, Rag2$^{-/-}$ Itk$^{+/-}$, and Rag2$^{-/-}$ Itk$^{-/-}$ littermates for ILC2 in the intestinal tissue. We reasoned that in these mice, lacking all T cells and B cells, we would eliminate any environmental alterations due to the deficiency of ITK in the T cell compartment. These data are now shown in Fig. 5A and B in the revised manuscript. As shown, in the Rag-deficient background, ILC2 frequencies are reduced in the absence of ITK. These results argue for a cell-intrinsic requirement for ITK in intestinal ILC2 homeostasis even in the absence of T cells.

6. Labeling of Fig. 8 is not correct (E is shown twice). Furthermore, in Fig. 8E and 8F all histograms are labeled with "siLP". According to the legend cLP ILC2 should be displayed as well.

We thank the reviewer for pointing out this error. The figure labels have been corrected in the revised manuscript.

7. IL-2c treatment alters T cell homeostasis in mice (Boyman *et al*., 2006). Does the expansion of intestinal ILC2 in IL-2c-treated *Itk*$^{-/-}$ mice result from a normalization of T cell homeostasis (allowing the restoration of IL-2-independent T-ILC2 interactions) or does it result from the direct action of IL-2c on ILC2? This question should be addressed experimentally, especially because the authors "speculated that the availability of IL-2 from T cells in the gut microenvironment of *Itk*$^{-/-}$ mice would likely be limited leading to disruption of ILC2 tissue homeostasis." To support this point, experiments analyzing intestinal *Itk*$^{-/-}$ ILC2 in a normal T cell-competent environment (see mixed BM chimeras above) are required.

We agree with the reviewer's opinion that IL-2c treatment could cause changes in T cells, thereby indirectly affecting the restoration of the ILC2 population. The clone of antibody that we used for the IL-2 complex injections was JES6-1A12, which is known to preferentially bind to CD25 on Treg (Webster et al., 2009)*, while a different clone, S4B6, binds preferentially to CD122 on conventional T cells* (Boyman et al., 2006;

Spangler et al., 2015). *Due to this, we expected IL-2c injections in our experiments might affect Treg and/or ILC2, but have little effect on conventional T cells. To verify this, we examined T cell populations in intestinal tissue after IL-2c injections. We observed that IL-2c complex injections led to modest increases in the numbers of Treg in the colon of both WT and Itk$^{-/-}$ mice. Also, as expected, we saw no significant changes in overall CD4^{+} T cell numbers in the colon of IL-2c-injected WT or Itk$^{-/-}$mice. These data have now been included in the revised manuscript as Figure S7.*

References

Boyman, O., Kovar, M., Rubinstein, M.P., Surh, C.D., Sprent, J., 2006. Selective stimulation of T cell subsets with antibody-cytokine immune complexes. Science 311, 1924–1927. doi:10.1126/science.1122927

Spangler, J.B., Tomala, J., Luca, V.C., Jude, K.M., Dong, S., Ring, A.M., Votavova, P., Pepper, M., Kovar, M., Garcia, K.C., 2015. Antibodies to Interleukin-2 Elicit Selective T Cell Subset Potentiation through Distinct Conformational Mechanisms. Immunity 42, 815–825. doi:10.1016/j.immuni.2015.04.015

Webster, K.E., Walters, S., Kohler, R.E., Mrkvan, T., Boyman, O., Surh, C.D., Grey, S.T., Sprent, J., 2009. In vivo expansion of T reg cells with IL-2-mAb complexes: induction of resistance to EAE and long-term acceptance of islet allografts without immunosuppression. The Journal of Experimental Medicine 206, 751–760. doi:10.1084/jem.20082824

8. The authors measured Annexin V levels to visualize cell death after IL-2c treatment (Fig. 8E). How do the authors explain that death rates of intestinal ILC2 do not differ between untreated WT and *Itk*$^{-/-}$ mice although ILC2 numbers are strongly reduced in the latter?

The reviewer points out an interesting but surprising finding. We can only speculate that the Itk$^{-/-}$ ILC2 in untreated mice are rapidly cleared from the tissue by resident macrophages or other phagocytic cells, whereas in the IL-2c treated mice, these cells accumulate faster than the clearance mechanism can remove them. We have added a sentence describing this speculation to p. 22 of the revised manuscript.

Reviewer #3 Comments

In "The Tec kinase ITK promotes intestinal tissue integrity via regulation of ILC2", Cho and her colleagues examine the role of the Tec kinase, Itk, in the homeostasis of ILC2's in the gut. They report that whole animal deletion of *Itk* is associated with significant decrease in numbers of ILC2's, but only in the small intestinal lamina propria. They find no evidence for decreased expression of intestine-specific chemokine or integrin receptors, or failure to respond to IL-33. They note that this is associated with disrupted intestinal integrity with increased permeability and increased morbidity and mortality following DSS colitis. There is a significant effort made to determine if there is a developmental defect that inhibits migration into the BM; however, the BM compartment looks normal. Instead, the authors suggest the ILC2 defect is secondary to a decrease in the number of conventional T cells and a lack of IL-2. Thus, they show that treatment of *Itk*-deficient mice with IL-2-anti-IL-2 complexes rescues the compartment.

The manuscript is easy to read and the figures are quite clear.

This is a very interesting observation. However, my primary concern is that it doesn't explain the tissue specificity of the ILC2 deficit.

We appreciate the reviewer's comment. We agree with the comment that the mechanism of tissue-specific ILC2 defect in Itk$^{-/-}$ mice has not explained in the manuscript. However, in the revised manuscript, we added our experiment to examine the status of lung T cell population in Itk$^{-/-}$ mice in the steady-state conditions. As shown in Figure S7F and G, lung T cells in Itk$^{-/-}$ mice did not exhibit the defect as gut T cells did in the intestine arguing that the possibility that the mode of tissue-specific phenotype is caused by T cell availability in the intestine. Also, Itk mRNA expression is reported to be constant regardless of tissue origins (Ricardo-Gonzalez et al., 2018). This also excludes the possibility that varied ITK expression affects tissue-specific ILC2 homeostasis.

1. Do the ILC2's in other tissues express ITK?

According to a recent paper from R. Locksley and colleagues, ILC2 isolated from skin, gut, lung, fat, and bone marrow all express comparable amounts of Itk mRNA (Ricardo-

Gonzalez et al., 2018).*This statement and the accompanying reference have been added to the revised manuscript on p. 6.*

Reference

Ricardo-Gonzalez, R.R., Van Dyken, S.J., Schneider, C., Lee, J., Nussbaum, J.C., Liang, H.-E., Vaka, D., Eckalbar, W.L., Molofsky, A.B., Erle, D.J., Locksley, R.M., 2018. Tissue signals imprint ILC2 identity with anticipatory function. Nat. Immunol. 19, 1093–1099. doi:10.1038/s41590-018-0201-4

2. Are there normal numbers of conventional T cells in other tissues? If yes, this would explain the intestine specificity. If not, then it is not the explanation. Does infection not increase the number of local T cells? Or is the increase not adequate to generate IL-2?

We have checked T cells in the lungs of WT and Itk$^{-/-}$ mice under steady-state conditions. Unlike the situation in the intestine, Itk$^{-/-}$ mice have no reduction in steady-state numbers of T cells in the lung. These data are now included in the revised manuscript in Figure S7F and G. We also include a comment indicating that the steady-state numbers of T cells correlates with steady state ILC2 numbers in each tissue.

3. The authors suggest that the phenotype is cell intrinsic, given the similar finding in transfers to *Rag*-deficient mice. If however, the effect is due to inadequate T cell IL-2, can it be corrected with normal T cells? I can think of two ways to ask this question.

1) If *Itk*$^{-/-}$ BM progenitors are transferred into mice with WT T cells, do normal numbers end up in the small intestine?

We thank the reviewer for this suggestion. We have considered how to do this experiment. We were unable to find published studies in which ILC reconstitution in the intestine by adoptive transfer was performed in WT recipients (with WT intestinal T cell numbers). Instead, ILC adoptive transfers are generally done into a lymphopenic mouse strain, such as Rag$^{-/-}$ Il2rg$^{-/-}$. We were concerned that co-adoptive transfer of Itk$^{-/-}$ILC2 with WT IL-2 producing CD4^{+} T cells would generate a substantial homeostatic proliferation response that would complicate the interpretation of the data. Further, this protocol could also lead to systemic inflammation in the colon (such as in Fiona Powrie's T cell adoptive transfer colitis model), also complicating the interpretation of

the data.

2) A cleaner experiment: in a mixed BM chimera, are the intestinal ILC2's equally WT and *Itk*-deficient?

As described above in the response to reviewer #2's comments, our effort to generate BM chimeras encountered technical difficulties as few mice survived the procedure. As an alternative, we show data from Rag$^{-/-}$versus Rag$^{-/-}$Itk$^{-/-}$ littermates in the revised manuscript in Fig. 5A and B.

4. Given the phenotype of DSS, as well as, the intestinal permeability defect, do *Itk*-deficient ILC2s express normal levels of amphiregulin? Is that concretely what's lacking in the intestine?

We have examined amphiregulin production by intracellular staining of intestinal ILC2 from WT and Itk$^{-/-}$ mice stimulated ex vivo. These data show reduced amphiregulin production by Itk$^{-/-}$ ILC2 compared to WT ILC2, findings that are consistent with the exacerbated weight loss and impaired recovery of Itk$^{-/-}$ mice treated with DSS. These data are now included in the revised manuscript in Figure 6C and D.

5. The authors point out that the ILC2s that make it to the intestine have normal levels of IL-33R and other receptors. However, this would be expected for those cells that do home there - the difficulty is in determining the phenotype of those that don't.

We agree with the reviewer's comment. The expression of gut-homing receptors and cytokine receptors (IL-25R and IL-33R) on WT and Itk$^{-/-}$ gut ILC2 showed no significant differences either in the steady-state or in the adoptive transfer experiments. In the case of the adoptive transfer experiments, we were able to examine gut-homing/retention receptors integrin α4β7 and CCR9 on transferred ILC2 from the spleen and mLN of recipients. These data, now shown in Figure S6, did not reveal any reduced expression of these receptors by Itk$^{-/-}$ versus WT cells.

6. Where does the death occur in the absence of IL-2? Do cells not make it to the intestine? Do they enter with normal chemokine receptor and integrin expression and then die? (The latter is what is implied by the interpretation preferred)

Based on the expression of gut-homing receptors, we speculated that the migration to intestine of ILC2 is not affected by the Itk deficiency. We have not examined the cell death process in vivo or the kinetics of cell death in the intestine after the transfer of cultured Itk$^{-/-}$ ILC2 precursors. However, based on the substantial T cell deficit in the intestine, we assume that IL-2 availability will primarily be limiting in the intestine, and consequently, that it is in this tissue that the homeostasis of ILC2 is primarily affected.

REVIEWERS' COMMENTS:

Reviewer #2 (Remarks to the Author):

The revised manuscript has improved a lot and my points were addressed satisfactorily. However, prior to publication the following points must be addressed:

1. Figure legends in general: The authors should always indicate how many mice were analyzed and how many independent experiments were performed. In some legends this information is missing.
2. Legend Fig. 4 (F): The authors state “Compiled MFIs from three independent experiments are shown (F).“ According to the Source Data file only 2 experiments were performed.
3. Legend Fig. 4 (I): The authors state “Data were compiled from three independent experiments.“ According to the Source Data file only 2 experiments were performed.
4. Please clarify why some of the values related to Fig. 2F and Fig. 2H (see Source Data files) are identical although different parameters were analyzed. The same is true for the values related to Fig. 5D and Fig. 5F.

Reviewer #3 (Remarks to the Author):

The authors did an outstanding job of responding to the reviewers' concerns and altering their manuscript appropriately.

I note that two reviewers thought that mixed bone marrow chimeraes would be very useful to verify the proposed mechanism. If they can't be made, is there a way to acknowledge their utility?

Point-By-Point Response to Reviewers

We thank the reviewers again for their critical comments on our revised manuscript. Below, please find our response to each comment in a point-by-point manner. Our response to the reviewer's comments are all in blue italic font.

Reviewer #2 (Remarks to the Author):

The revised manuscript has improved a lot and my points were addressed satisfactorily. However, prior to publication the following points must be addressed:

1. Figure legends in general: The authors should always indicate how many mice were analyzed and how many independent experiments were performed. In some legends this information is missing.

We thank the reviewer for pointing out our inconsistency in describing the number of experiments and mice used in each figure. We have added an appropriate statement to each figure legend with this information.

2. Legend Fig. 4 (F): The authors state "Compiled MFIs from three independent experiments are shown (F)." According to the Source Data file only 2 experiments were performed.

Thank you for pointing out this error. We corrected our statement in the legend to Figure 4.

3. Legend Fig. 4 (I): The authors state "Data were compiled from three independent experiments." According to the Source Data file only 2 experiments were performed.

The legend to Figure 4 was corrected.

4. Please clarify why some of the values related to Fig. 2F and Fig. 2H (see Source Data files) are identical although different parameters were analyzed. The same is true for the values related to Fig. 5D and Fig. 5F.

We thank the reviewer for pointing out this critical error. We have added the right data set information to the Source Data file to accurately match the data presented Fig. 2F and H and Fig. 5D and F.

Reviewer #3 (Remarks to the Author):

The authors did an outstanding job of responding to the reviewers' concerns and altering their manuscript appropriately.

I note that two reviewers thought that mixed bone marrow chimeras would be very useful to verify the proposed mechanism. If they can't be made, is there a way to acknowledge their utility?

Please find Supplementary Figure 6 in our revised manuscript. We added the survival graph of BM chimera recipients to acknowledge the limitation of their use. We have also included a sentence (p.14 of revised manuscript text) to indicate the potential utility of this information.
